# Early immune suppression leads to uncontrolled mite proliferation and potent host inflammatory responses in a porcine model of crusted versus ordinary scabies

**DOI:** 10.1371/journal.pntd.0008601

**Published:** 2020-09-04

**Authors:** Sajad A. Bhat, Shelley F. Walton, Tomer Ventura, Xiaosong Liu, James S. McCarthy, Stewart T. G. Burgess, Kate E. Mounsey

**Affiliations:** 1 School of Health & Sport Sciences, University of the Sunshine Coast, Sippy Downs, Queensland, Australia; 2 Animal and Bioscience Research Department, Teagasc, Grange, Ireland; 3 Genecology Research Centre, University of the Sunshine Coast, Sippy Downs, Queensland, Australia; 4 Cancer Research Institute, The First People’s Hospital of Foshan, Foshan, Guangdong, China; 5 Infectious Diseases Division, QIMR Berghofer Medical Research Institute, Brisbane, Queensland, Australia; 6 Diagnostics, Moredun Research Institute, Pentlands Science Park, Bush Loan, Edinburgh, United Kingdom; Hitit University, Faculty of Medicine, TURKEY

## Abstract

Scabies is a neglected tropical disease of global significance. Our understanding of host-parasite interactions has been limited, particularly in crusted scabies (CS), a severe clinical manifestation involving hyper-infestation of *Sarcoptes scabiei* mites. Susceptibility to CS may be associated with immunosuppressive conditions but CS has also been seen in cases with no identifiable risk factor or immune deficit. Due to ethical and logistical difficulties with undertaking research on clinical patients with CS, we adopted a porcine model which parallels human clinical manifestations. Transcriptomic analysis using microarrays was used to explore scabies pathogenesis, and to identify early events differentiating pigs with ordinary (OS) and crusted scabies. Pigs with OS (n = 4), CS (n = 4) and non-infested controls (n = 4) were compared at pre-infestation, weeks 1, 2, 4 and 8 post-infestation. In CS relative to OS, there were numerous differentially expressed genes including pro-inflammatory cytokines (IL17A, IL8, IL19, IL20 and OSM) and chemokines involved in immune cell activation and recruitment (CCL20, CCL27 and CXCL6). The influence of genes associated with immune regulation (CD274/PD-L1 and IL27), immune signalling (TLR2, TLR8) and antigen presentation (RFX5, HLA-5 and HLA-DOB) were highlighted in the early host response to CS. We observed similarities with gene expression profiles associated with psoriasis and atopic dermatitis and confirmed previous observations of Th2/17 pronounced responses in CS. This is the first comprehensive study describing transcriptional changes associated with the development of CS and significantly, the distinction between OS and CS. This provides a basis for clinical follow-up studies, potentially identifying new control strategies for this severely debilitating disease.

## Author summary

The immune response to *Sarcoptes scabiei* infestation is poorly defined. There have been few studies of crusted scabies, a debilitating clinical variant of the disease characterised by extremely high mite numbers. In this study, we used a pig model to explore differences in gene expression between clinical variants of scabies, including a focus on immune events occurring prior to the development of clinical signs. In early infestation, genes relating to inflammation, immune recognition and cell migration were potently suppressed in pigs with crusted scabies. This suggests that these pigs lacked the ability to mount a timely, effective immune response, allowing mites to proliferate unchecked. In later infestation, the large numbers of mites then triggered a strong inflammatory response leading to severe skin pathology. Gene expression profiles in crusted scabies shared similarities with other inflammatory skin diseases such as psoriasis. This is the first study to compare immune responses in crusted and ordinary scabies in early infestation and reveals new insights into the progression of disease. Findings may lead to the development of new approaches to diagnose and treat this important, but neglected disease.

## Introduction

In scabies, a wide range of clinical features are recognized, from Ordinary scabies (OS), to the rare and destructive Crusted or Norwegian scabies (CS). In OS, the host skin generally shows a low parasite burden (<20 mites) and is associated with some features of an allergic type skin reaction with intense generalized pruritus. In contrast, CS is a debilitating clinical variant characterized by hyper-infestation of mites (thousands per gram of skin) and the development of hyperkeratotic skin crusts [1]. The extremely high mite burden in CS makes it more infectious, and the severe skin damage increases the risk of serious secondary bacterial sepsis. As a consequence CS can contribute to ongoing high community prevalence of scabies and challenge community management programs [2]. Scabies has been formally recognized by the WHO as a neglected tropical disease, and indeed CS is an even more neglected aspect of this disease that warrants further study.

Crusted scabies is caused by the same variant of *Sarcoptes scabiei* mites as those causing OS [3] indicating that increased mite virulence does not cause CS. Re-infestation is common in patients with CS, whereas in OS mite numbers reduce with repeat infestation suggesting the development of protective immunity [4]. Specific risk factors for development of CS are poorly understood. Since its first recognition in patients with leprosy [5] it has been observed that immunosuppression is a predisposing factor associated with CS. Other predisposing conditions include human immunodeficiency virus, human T-lymphocytic virus 1 and iatrogenic immune suppression (e.g. for organ transplantation and cancer chemotherapy). Furthermore, CS has been reported in developmental disability, including Down syndrome [4] and institutional outbreaks of OS resulting from index CS cases are relatively common [6, 7]. Notably CS is also observed in patients with no identified risk factor or immunodeficiency [5]. From these reports, it seems that the susceptibility of this cohort to CS may be due to host immune suppression and/or dysregulation, the nature of which is not yet clear.

Different host immune responses have been observed in OS and CS [as reviewed in 8]. In OS, the immune response is reportedly dominated by a Th1-type cytokine profile, with CD4^+^ T cells being the most prevalent T lymphocytes in the skin [9]. In contrast, CS resembles a non-protective allergic response with elevated Th2 and Th17 cytokine profiles including interleukin (IL) 4, IL-5, IL-13 and IL-17, extremely high immunoglobin (Ig) E levels, and CD8^+^ T lymphocytes as the predominant effector cells in the skin [1, 10–12]. The reasons for these differential immune responses have not been identified. Observations of host responses in CS so far are collated from a small number of cross-sectional humoral and cellular studies, as opportunities for longitudinal study of human infestation are limited. In this study, we used a novel porcine model with clinical manifestations resembling human scabies [13] to compare gene expression profiles between pigs with CS and OS. Our hypothesis was that these clinical manifestations will be reflected by dramatic differences in gene expression within these hosts. We were also interested in gene expression prior to infestation to gain insights into any underlying susceptibility in the absence of infestation and to possibly identify the specific immune factors predisposing the hosts to CS. These early events may provide critical information about the regulators of disease progression and development.

## Materials and methods

### Ethics statement

The study was approved by the Animal Ethics Committees of the University of the Sunshine Coast (Approval number AN/A/13/71), the QIMR Berghofer Medical Research Institute (Approval number P1266) and the Queensland Department of Agriculture, Forestry and Fisheries (Approval number SA/2013/02/416). All animals were handled in strict accordance with good animal practice as defined by the Australian code of practice for the care and use of animals for scientific purposes.

### Experimental *S*. *scabiei* infestation and clinical monitoring

The work presented herein was a subset of a larger experimental trial undertaken at the Queensland Agricultural Science Precinct (QASP), University of Queensland, Gatton QLD, Australia [11]. For the overall study, 18 three-week-old piglets (*Sus scrofa*) of the large white breed were used. These pigs had been randomly allocated to mite infested (n = 12) or non-infested control (n = 6) groups. In the infested group, the ears of the pigs were inoculated with *Sarcoptes scabiei var*. *suis* mites (approximately 200 of mixed developmental stages) obtained from our existing mange pig model. The infested and non-infested pigs were housed separately in identical rooms to avoid accidental transmission of mites. The rooms were maintained at a constant temperature of 24°C and provided with a 12-hour photoperiod. The experimental infestations and housing protocols were as described previously [11, 13]. At the conclusion of the overall study pigs were euthanized, inspected by a veterinarian post-mortem, and additional blood, skin and tissue samples collected.

Pigs were monitored on a weekly basis for disease progression. As described previously [11, 13] the severity of the skin lesions was scored on a scale from 1–8 (where 1 = mild papular rash, 2–4 = papular rash of increasing intensity, accompanied by exudates and increasing inflammation, >4 = development of hyperkeratotic lesions of increasing area, 8 = severe hyperkeratosis with development external to ears). In this study, CS was categorized as a lesion score of ≥4 at more than one time point during the trial, and OS was categorized as a lesion score of <4 across all time points. Significant differences between lesion scores of pigs designated CS and OS were measured using a two-way repeated measures ANOVA in GraphPad Prism.

### Sample collection and RNA extraction

Skin samples for RNA extraction from all animals were collected using sterile disposable 3.5 mm biopsy punches (McFarlane Medical, Surrey Hills, Australia). Biopsies were collected from the center of lesions in the ear where scabies lesions were apparent, and from similar areas of the ear in non-infested pigs. The average weight of skin biopsies was 10 milligrams. Biopsies were stored in 1 ml of RNA Later (Qiagen, Victoria Australia), transported on ice and stored at -80°C until processing.

Samples for microarray were selected retrospectively based on clinical scores of individual pigs during the trial. Comparison groups were CS (n = 4), OS (n = 4) and non-infested controls (n = 4). Although the overall study period was 16 weeks, we focused on the first 8 weeks to identify changes related to disease susceptibility early in the infestation prior to the development of high mite burdens and severe clinical pathology. Total RNA was extracted from the skin biopsies at pre-infestation (week 0), and weeks one, two, four and eight post-infestation (wpi), giving a total of 60 samples. Skin biopsies in RNA later were thawed on ice, and cut into smaller pieces using a sterile scalpel blade. The skin biopsy pieces were then homogenized in 1mL of TRIzol reagent (Life Technologies, Victoria, Australia) using a Minilys Homogenizer with CK 28 ceramic beads (Bertin Instruments, Bretonneux, France). Phase separation with TRIzol was undertaken according to the manufacturers’ instructions. The aqueous phase was column purified using the DirectZol RNA MiniPrep kit (Zymo Research, Integrated Sciences, New South Wales, Australia) as per the manufacturers’ protocol and on-column DNAse digestion was carried out using PureLink DNase (Life Technologies). RNA was eluted in 50 μL nuclease free dH_2_0 and stored at -80°C. RNA sample integrity was assessed on a 2100 Bioanalyzer System (Agilent Technologies, Inc., California, USA), and RNA concentration was quantified using a ND-2000 NanoDrop spectrophotometer (Thermo Scientific, Delaware, USA). RNA samples with an RNA integrity number (RIN) >7.0 were considered to be of acceptable quality [14].

### Microarray

Microarray analysis was performed using the A-GEOD-16571 Agilent Porcine Gene Expression Microarray V2 4x 44K platform containing 43,803 probe sets from *Sus scrofa*. The One-Colour Microarray Based Gene-Expression workflow using Low Input Quick Amp Labelling and RNA Spike-In Kit (Agilent) was used to amplify and process the total RNA, following the manufacturer’s recommended protocols. The recommended amount (1.65 μg) of each cRNA sample was processed at the Ramaciotti Centre for Genomics, (Sydney NSW) following the manufacturers’ protocol. Samples from all animals in a group (from either infected or non-infected) at each time point were hybridized onto the arrays on a single slide to limit technical variation. Two slides were used for the infested groups and a single slide for the non-infested group at each time point across a total of 15 slides (60 arrays in total). Microarrays were scanned on a microarray scanner (Agilent) at the manufacturers’ recommended settings. Feature Extraction software version 10.7.3 (Agilent) was used to extract data signals from the probe features on the arrays. Quality control analysis by reviewing the control information from all arrays was carried out to ensure quality and consistency of sample labelling and array hybridizations. Feature extraction data were then imported into Partek Genomics Suite Version 6.5 for downstream filtering and statistical analysis. To explore the preliminary data, box and whisker visualizations, histogram plot and principal component analysis (PCA) were carried out to assess the distribution profile of the dataset, identify outliers, sample to sample variation and to assess relationships between samples. Quantile Normalization [15] of the raw expression data was carried out to normalize the distribution of probe fluorescence intensities among different arrays and the data were log transformed.

### Assessment of differential gene expression

Differential gene expression was determined using a two way-analysis of variance (ANOVA) with a Fisher's Least Significant Difference (LSD) post-hoc test. Crusted scabies (CS, n = 4) were compared to OS (n = 4) samples at zero (pre-infestation), one, two, four and eight wpi. Analysis was also undertaken for CS (n = 4) vs Control (C, n = 4); and OS (n = 4) vs C (n = 4) at each time point. Multiple test correction was performed to generate the gene lists using the False Discovery Rate (FDR) [16] procedure with an FDR corrected p-value threshold of ≤0.05 and a fold change (FC) threshold of ≥± 2.0.

### Annotation of differentially expressed genes

From the resulting gene lists, it was evident that many probes (70–75%) which were differentially expressed were not annotated on the Agilent Array annotation file. The probes were subsequently annotated by individual BLAST analysis of the EST sequences represented by each probe to *Sus scrofa* cDNA using genome assembly Sus_scrofa.scrofa10.2.cdna.all.fa.gz (Ensembl) in CLC Genomics Workbench (Version 8.5.1, Qiagen). For filtering, stringent parameters of greater than 40 base pair (average base pair length of array probes was 60) match, ≥ 95% identity and an E-value cut off of 1.00E-5 were applied. The pig cDNA was then annotated to coding sequences (CDS) of *Homo sapiens* cDNAs using genome assembly GRCh38.cds.all (Ensembl) in CLC Genomics Workbench. For filtering, stringent parameters of a >200 base pair match, ≥ 60% identity (as alignments were made against the coding regions) and an E-value cut-off of 1.00E-5 were applied. Gene symbol (HUGO Gene Nomenclature Committee, HGNC) level annotation was used and the relevant homologous human gene symbol was used where the relevant porcine annotation was unavailable.

### Data accession

All the microarray data is Minimum Information about a Microarray Experiment (MIAME) compliant [17]. Protocols of the experimental procedures, sample information, methods of analysis and microarray data (array information, raw data, and processed data) are available as supplementary information in the European Bioinformatics Institute’s ArrayExpress database http://www.ebi.ac.uk/arrayexpress (accession number E-MTAB-6433).

### Identification of signalling pathways and gene networks involved in the host response to scabies infestation

Gene lists from each of the individual comparisons were uploaded into Ingenuity Pathway Analysis (IPA, Ingenuity Systems, Qiagen) and a network/pathway analysis was performed. Each gene identifier was mapped to its corresponding gene object in Ingenuity’s Knowledge Base. Gene networks (graphical visualizations of the molecular relationships between the genes) were created based on their connectivity with the genes in the input data. According to how relevant they are to the genes in the dataset, each network was assigned a Z-score and a right tailed Fisher’s exact test was used to calculate a p-value for each network. Canonical pathway analysis was performed to identify the biological pathways associated with the molecules in the input dataset. The significance values (p-value) of the association of genes from the dataset with each canonical pathway was calculated using a right tailed Fisher’s exact test. Gene lists derived from OS vs C and CS vs C groups at weeks 1 and 8 post-infestation were used as input into Venny (Version 2.1, BioinfoGP Service, http://bioinfogp.cnb.csic.es/tools/venny/index.html) to generate Venn diagrams to further identify shared and unique differentially expressed genes (DEGs) between these clinical manifestations. The functions of the DEGs were confirmed from IPA, GeneCards (http://www.genecards.org/) and Uniprot (http://www.uniprot.org/) online databases.

### qRT-PCR validation

Quantitative real-time PCR (qRT-PCR) was used to validate the gene expression results obtained from the microarray analysis. Eight DEGs in the 2-way ANOVA analysis with FDR corrected p-value of ≤ 0.05 and FC of > 2.0 were selected and evaluated by qRT-PCR. One microgram of total RNA was reverse transcribed into complementary DNA (cDNA) in duplicate using the QuantiTect Reverse Transcription Kit (Qiagen) following the manufacturers’ protocol. Primers (S1 Table) were either as previously described [12, 18, 19] or newly designed using Primer-BLAST (https://www.ncbi.nlm.nih.gov/tools/primer-blast/). The porcine hypoxanthine phosphoribosyl transferase 1 (HPRT1) gene was used as a housekeeping control [12, 20]. qRT-PCR reactions were carried out using the QuantiTect SYBR green PCR kit (Qiagen) as previously described [12]. Relative quantification of gene expression levels was determined by normalizing to the HPRT1 control using the Comparative Ct method [21] and expressed as fold change.

## Results

### Clinical progression of mange infection in pigs

**Fig 1:**
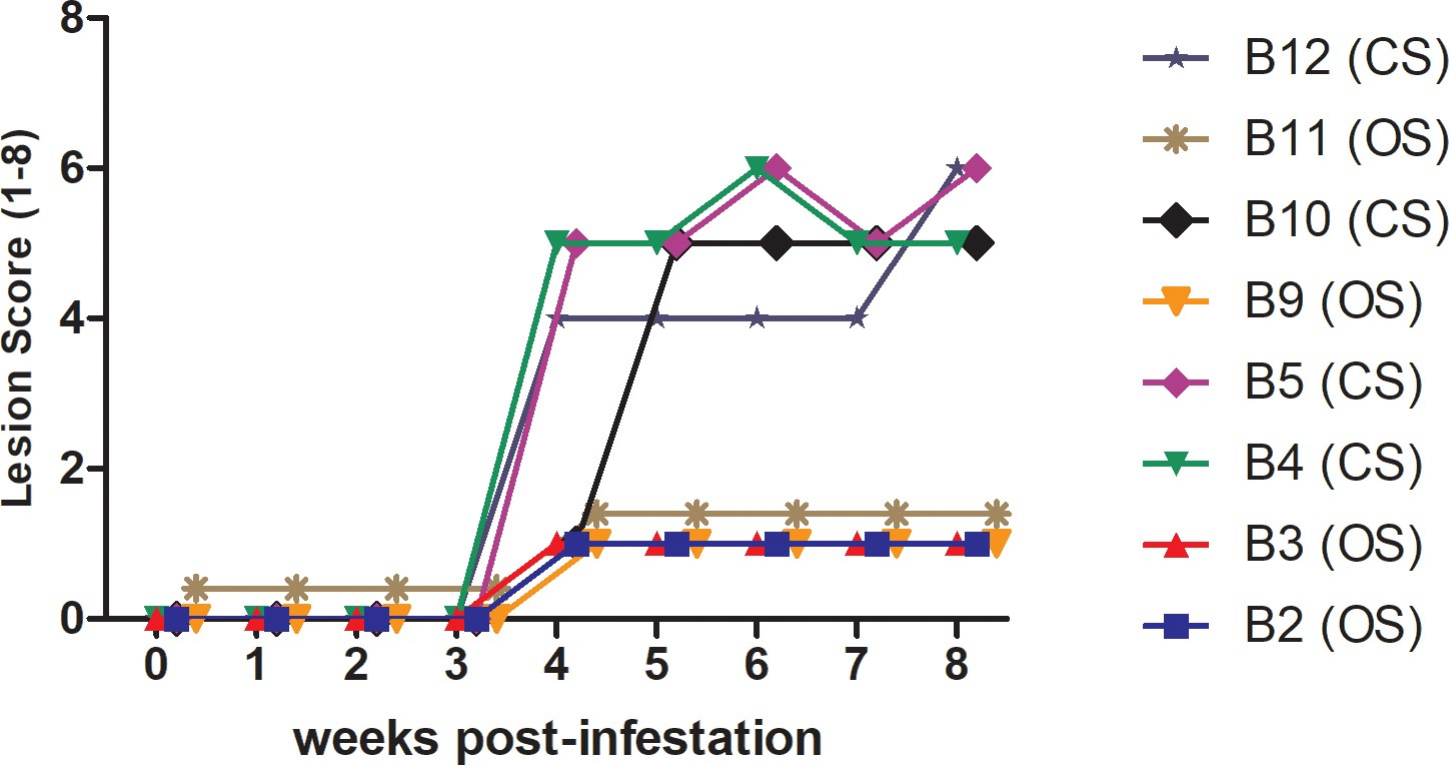
Clinical score and lesion progression in mite infested pigs (n = 8). Ear lesions were scored weekly. Score of 1–4: acute mange with generalized rash and papular lesions of increasing density, 4: development of increasing encrustment, 8: extensive encrustment spreading externally to the ears. Significant difference in lesion score between pigs classified as CS vs OS was observed from Week 4 onwards (p < 0.0001).

Based on previous studies using the porcine model [13, 22] a range of clinical manifestations were expected in infested pigs. The non-infested pigs did not show skin lesions at any time during the trial. Pigs in the infested group began to exhibit clinical signs of lesion development from 4 wpi. Based on clinical scores, by 8 wpi, five infested pigs were classified as OS (score < 4) and seven pigs were classified as CS (score ≥ 4). The difference in lesion scores between pigs classified as CS and OS was significant from week 4 onwards (p < 0.0001). From these, four pigs with CS and four with OS, selected retrospectively at random were included in the microarray analysis (Fig 1). Four pigs were also randomly selected from the non-infested control group (C).

### Microarray data processing and quality control

**Fig 2:**
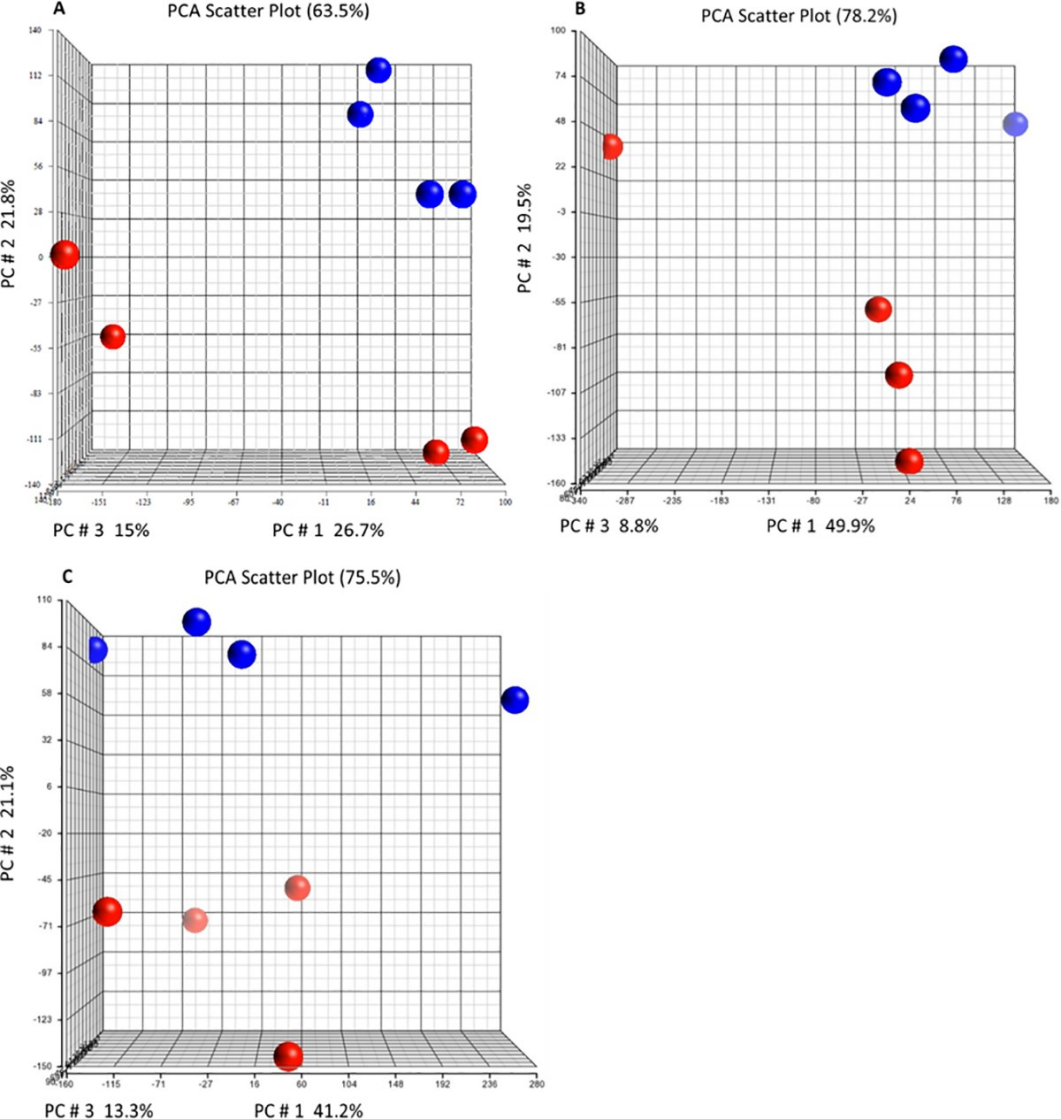
Principal component analysis (PCA) plot comparing gene expression in CS (n = 4) with OS (n = 4) samples. Samples from week 0 pre-infestation (A), and week 1 (B) and week 8 (C) following *S*. *scabiei* infestation are plotted. PCA demonstrated that the CS samples grouped separately to OS samples. Each circle represents 1 sample. Red circles indicate CS samples and blue indicate OS samples.

Good RNA yields were obtained at all time points with a mean RIN of 7.75 and optical density (OD) A_260/A280_ ratios between 1.8 and 2.1 indicating high quality RNA with minimal degradation. Likewise, the resulting labelled complementary RNA (cRNA) samples passed QC with yields and specific activity above the recommended levels and mean A_260/A280_ values of 2.10 demonstrating high-quality cRNA. The quality of array data was found to be consistent with the manufacturer’s recommendations, with box and whisker visualizations and histogram plots from the preliminary analysis confirming the data had comparable distributions and were of sufficient quality for further analysis (S1 Fig). An initial exploratory principal component analysis (PCA) revealed that the samples from the mite infested animals grouped according to their clinical phenotype (CS or OS) at each time point of the study, suggesting that distinct relationships existed between the samples (Fig 2).

### Assessment of differentially expressed transcripts

Hierarchical clustering of data agreed with the PCA showing differential expression of genes in CS compared to OS samples. From the preliminary gene lists generated, gene symbol level annotation from the manufacturer array was only available for 25–30% of significantly DEGs. Further gene annotation was achieved for a further 40% of probes, for a final overall annotation of approximately 65%. The complete gene lists for all comparisons are available at http://dx.doi.org/10.25907/5d2fbcaf59c5d.

A large number of genes were differentially expressed between pigs with CS and OS at each time point of the study with the exception of 4 wpi. At 1, 2 and 8 wpi a trend of significant downregulation was observed in CS relative to OS, with up to 80% of all DEGs downregulated in CS. At 4 wpi, a different trend was observed compared to other time points with almost all genes (259) upregulated and only two genes downregulated in CS (Table 1).

**Table 1:** Number of differentially expressed genes (DEGs) in CS vs OS following infestation with *S*. *scabiei* in pigs. Differential gene expression was deduced by 2-way ANOVA combined with a Fisher's Least Significant Difference (LSD) post-hoc test in CS (n = 4) relative to OS (n = 4) samples at time points 0 (pre-infestation) and 1, 2, 4 and 8 wpi. Number of up or down regulated genes with a p-value of ≤ 0.05 and fold change of ≥ ± 2.0 at each time point are indicated.

Time Point	Number of DEGs	Upregulated	Downregulated
**Pre-infestation** **(week 0)**	1297	613	684
**1wpi**	1305	99	1206
**2wpi**	1651	197	1454
**4wpi**	261	259	2
**8wpi**	1292	261	1031

**Fig 3:**
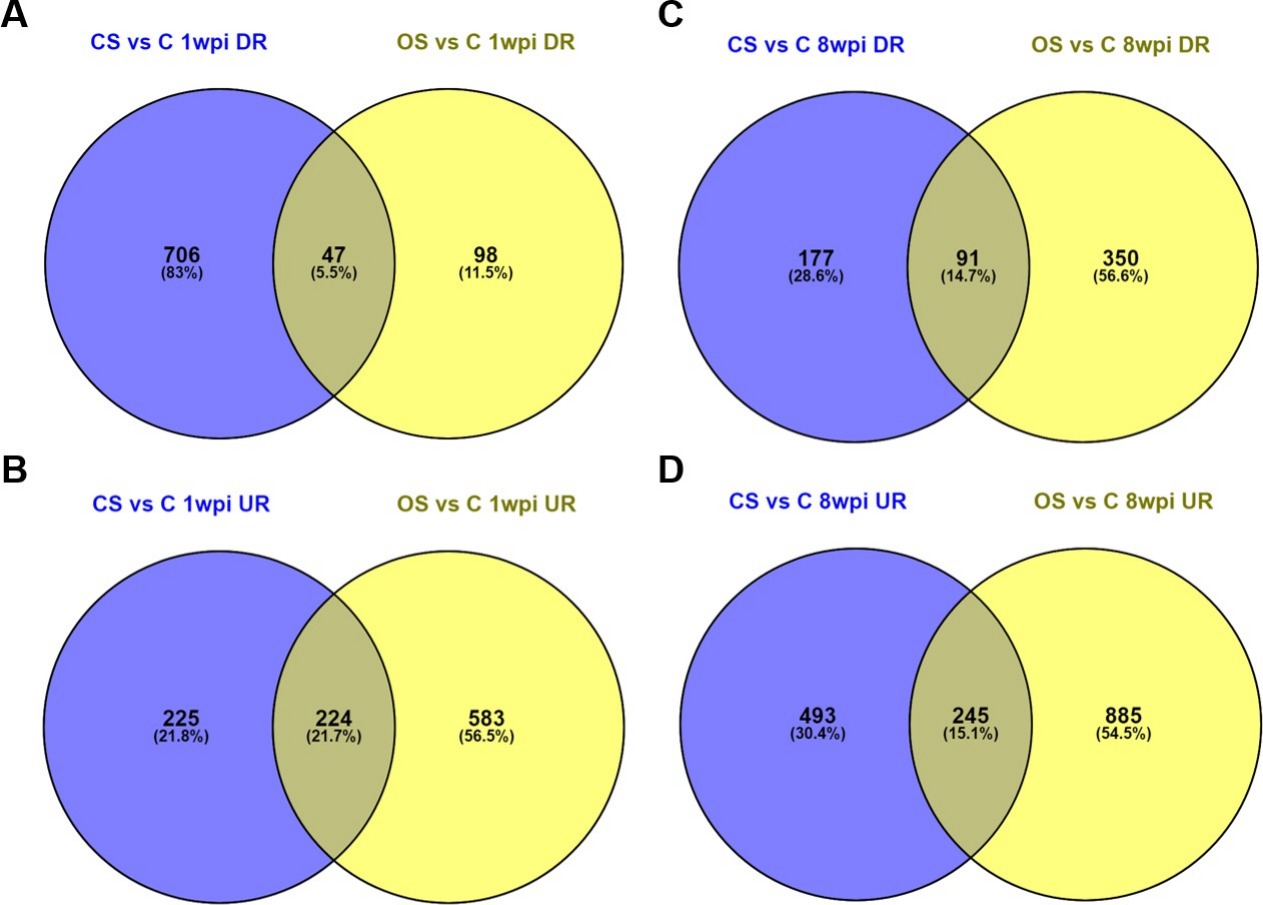
Venn diagrams showing shared and unique transcripts between CS vs C and OS vs C comparisons. Up (UR) and down (DR) regulated genes with a p-value <0.05 and fold change ≥ ± 2 were compared at 1 and 8 weeks post-infestation. A: week 1 downregulated genes, B: week 1 up regulated genes; C: week 8 down regulated genes and D: week 8 up regulated genes.

A comparison of DEGs in CS vs control and OS vs control pigs at 1 wpi (early, pre-clinical) and 8 wpi (late, clinical) was performed to identify shared and unique transcripts between CS and OS pigs (Fig 3). At 1 wpi in CS skin, 706 genes (83%) were found to be exclusively downregulated, whereas a smaller proportion were exclusively upregulated (n = 225 (22%)). Conversely at 1 wpi in OS, more genes were exclusively upregulated (n = 583 (57%)) than down regulated (n = 98 (11.5%)).

The top ten genes with the highest fold change (up and down-regulated) from each of the five time points of the analysis are shown in Table 2. Among those, highly differentially expressed genes in early CS included toll-like receptor 8 (TLR8, 44 fold at 1 wpi) and dopachrome tautomerase (DCT, -34 fold at 1 wpi, 18 fold at 2 wpi), associated with melanin synthesis. An anti-inflammatory adipokine (ADIPOQ) was downregulated (-24 fold) at 1 wpi. Later infestation (8 wpi) was characterized by strong upregulation of CXC motif chemokine ligand 6 (CXCL6, 64 fold), arginase (ARG1, 30 fold), and S100 calcium binding proteins (S100) A7 and A9 (21 fold). Further investigation of the gene lists by IPA showed that mite infestation in CS compared to OS animals resulted in the differential expression of an array of immune and proinflammatory mediators over the time course of the infestation (S2 Table).

**Table 2:** Top 10 differently expressed genes (DEGs) following infestation with *S*. *scabiei* in pigs. The DEGs included are those which showed ≥ ± 2 fold change in expression with p ≤ 0.05 in CS relative to OS pigs at time points week 0 (pre-infestation) and, 1, 2, 4 and 8 wpi. + is upregulated and—is downregulated.

Time Point	Gene Symbol	Gene Description	Fold Change
**Pre-infestation**	NAMPT	Nicotinamide phosphoribosyl transferase	+50.36
	SEC14L2	SEC14 like lipid binding 2	+50.02
	PLA2R1	Phospholipase A2 receptor 1	+49.34
	ARPC5L	Actin related protein 2/3 complex subunit 5 like	+37.77
	NPFFR1	Neuropeptide FF receptor 1	+36.45
	PDCD7	Programmed cell death 7	-22.9
	ZC3H15	Zinc finger CCCH-type containing 15	-22.38
	TDP1	Tyrosyl-DNA phosphodiesterase 1	-21.4
	PACSIN2	Protein kinase C and casein kinase substrate in neurons 2	-20.97
	NTNG2	Netrin G2	-18.09
**1wpi**	ACTN2	Actinin Alpha 2	+164.42
	TLR8	Toll like receptor 8	+44.47
	ZKSCAN5	Zinc finger with KRAB and SCAN domains 5	+40.9
	CRYGD	Crystalline gamma D	+36.38
	KIAA1468	KIAA1468	+32.26
	DCT	Dopachrome tautomerase	-33.75
	GTF3C3	General transcription factor IIIC subunit 3	-29.57
	ADIPOQ	Adiponectin, C1Q and collagen domain containing	-24.3
	CTTNBP2NL	CTTNBP2 N-terminal like	-18.06
	RPL8	Ribosomal protein L8	-15.2
**2wpi**	DCT	Dopachrome tautomerase	+18.1
	FAAH	Fatty acid amide hydrolase	+15.2
	CYB5B	Cytochrome B5 type B	+11.9
	NMUR1	Neuromedin U receptor 1	+9.08
	RBM38	RNA binding motif protein 38	+8.89
	ANXA2	Annexin A2	-17.41
	APP	Amyloid beta precursor protein	-16.87
	PLA2R1	Phospholipase A2 receptor 1	-16.72
	DENND4A	DENN domain containing 4A	-15.51
	HOXB13	Homeobox B13	-15.51
**4wpi**	SCGB1A1	Secretoglobin family 1A member 1	+62.13
	GLRX3	Glutaredoxin 3	+21.08
	WDR3	WD repeat domain 3	+15.12
	CBX3	Chromobox 3	+13.66
	RFX5	Regulatory factor X5	+11.7
	EMP1	Epithelial membrane protein 1	+11.2
	TXNDC11	Thioredoxin domain containing 11	+11.14
	SCP2	Sterol carrier protein 2	+10.5
	PNLIPRP1	Pancreatic lipase related protein 1	-3.73
	FAM63B	Family with sequence similarity 63 member B	-3.01
**8wpi**	CXCL6	C-X-C motif chemokine ligand 6	+64.32
	ARG1	Arginase 1	+30.79
	ADGRF1	Adhesion G protein-coupled receptor F1	+25.39
	S100A7	S100 calcium binding protein A7	+21.53
	S100A9	S100 calcium binding protein A9	+21.08
	PACSIN2	Protein kinase C and casein kinase substrate in neurons 2	-12.84
	CARMIL1	Capping protein regulator and myosin 1 linker 1	-12.67
	ADSS	Adenylosuccinate synthase	-10.71
	MFSD6	Major facilitator superfamily domain containing 6	-10.18
	CAPZA3	Capping actin protein of muscle Z-line alpha subunit 3	-9.74

### Gene network and pathway analysis of host response to *S*. *scabiei* infestation in CS versus OS pigs

Canonical pathway analysis showed the DEGs were associated with several notable signalling pathways over the time course of infestation. including“Acute Phase Response Signalling”, “T Cell Receptor Signalling”, “NF-κB Signalling”, “IL-12 Signalling and Production in Macrophages” and “Role of IL-17A in Psoriasis” (Table 3).

**Table 3:** Top 5 most significant (and top 5 immunity related) canonical signalling pathways from the IPA mapping at each time point following infestation with *S*. *scabiei* in CS vs OS pigs.

Time	Canonical Pathways	Ratio*	p-value
**Pre-infestation** **(week 0)**	Acute Phase Response Signalling	28/169	8.26E-07
	FXR/RXR Activation	23/126	1.40E-06
	LXR/RXR Activation	21/121	9.04E-06
	AMPK Signalling	28/189	7.79E-06
	IL-12 Signalling and Production in Macrophages	24/146	5.66E-06
	**Immunity related**		
	MIF Regulation of Innate Immunity	10/41	1.13E-04
	iNOS Signalling	10/44	2.12E-04
	Production of Nitric Oxide and Reactive Oxygen Species in Macrophages	24/193	5.23E-04
	Atherosclerosis Signalling	18/127	5.40E-04
**1wpi**	Axonal Guidance Signalling	54/450	1.13E-06
	Adipogenesis pathway	23/134	5.08E-06
	Thyroid Cancer Signalling	11/40	1.69E-05
	PPAR/RXR Activation	25/178	7.02E-05
	Human Embryonic Stem Cell Pluripotency	21/143	1.37E-04
	**Immunity related**		
	Role of Osteoblasts, Osteoclasts and Chondrocytes in Rheumatoid Arthritis	28/232	3.74E-04
	T Cell Receptor Signalling	16/109	8.28E-04
	STAT3 Pathway	12/73	1.33E-03
	PKCθ Signalling in T Lymphocytes	17/132	2.50E-03
	NF-κB Signalling	21/180	2.89E-03
**2wpi**	Acute Phase Response Signalling	38/169	9.49E-10
	G-Protein Coupled Receptor Signalling	43/272	3.42E-06
	cAMP-mediated Signalling	36/223	1.30E-05
	FXR/RXR Activation	23/126	6.80E-05
	TR/RXR Activation	19/98	1.24E-04
	**Immunity related**		
	IL-12 Signalling and Production in Macrophages	24/146	2.58E-04
	Role of MAPK Signalling in the Pathogenesis of Influenza	15/72	2.76E-04
	STAT3 Pathway	15/73	3.23E-04
	iNOS Signalling	10/44	1.40E-03
	Role of JAK family kinases in IL-6-type Cytokine Signalling	7/25	2.03E-03
**4wpi**	Nur77 Signalling in T Lymphocytes	6/59	8.03E-05
	Cytotoxic T Lymphocyte-mediated Apoptosis of Target Cells	4/32	5.88E-04
	Granzyme B Signalling	3/16	8.86E-04
	Mitochondrial Dysfunction	8/171	1.20E-03
	cAMP-mediated Signalling	9/223	1.70E-03
	**Immunity related**		
	iNOS Signalling	4/44	1.98E-03
	CTLA4 Signalling in Cytotoxic T Lymphocytes	5/99	7.25E-03
**8wpi**	LXR/RXR Activation	25/121	4.08E-08
	Aryl Hydrocarbon Receptor Signalling	25/140	7.71E-07
	Role of IL-17A in Psoriasis	7/13	3.31E-06
	T Cell Receptor Signalling	20/109	6.25E-06
	Glucocorticoid Receptor Signalling	37/287	8.55E-06
	**Immunity related**		
	Regulation of IL-2 Expression in Activated and Anergic T Lymphocytes	16/79	1.43E-05
	CD28 Signalling in T Helper Cells	20/131	9.83E-05
	IL-8 Signalling	26/197	1.21E-04

*Ratio = number of genes/total number of genes in the pathway, wpi = weeks post-infestation.

IPA network analysis revealed that the *S*. *scabiei* infestation affected diverse biological functions and cellular processes. Biological functions included “Cellular Growth and Proliferation”, “Cellular Movement”, “Cell-to-Cell Signalling”, “Gene Expression”, “Cell-mediated Immune Response”, and “Inflammatory and Allergic Response”. The DEGs were also associated with various diseases including “Immunological Disease”, “Inflammatory Disease”, “Haematological Disease”, “Organismal Injury and Abnormalities”, and “Connective Tissue Disorders”.

### Gene expression profiles pre-infestation

Many genes were differentially expressed prior to experimental mite challenge at week 0 (Table 1, S2 Table) suggesting inherent differences possibly predisposing pigs to the development of CS or OS. Top canonical pathways associated with the dataset at week 0 included Acute Phase Response Signalling (S2 Fig), FXR/RXR Activation and AMPK Signalling (Table 3).

Acute phase signalling pathways were predicted to be more active in CS pre-infestation, with upregulated acute phase protein genes including Haptoglobin (HP, 2.6 fold), Transferrin (TF, 9.7 fold), and Oncostatin M (OSM, 13.8 fold). Conversely, Serum Amyloid A was downregulated (SAA, -5.5 fold). Significant differences in macrophage related pathways were observed at pre-infestation, although a prediction for activation or inhibition of these pathways was not discernable. Differential gene expression related to innate immunity included Myeloid differentiation primary response protein (MYD88, 6.7 fold) and interleukin 12B (IL12B, 2.7 fold). The ‘CD27 signalling in lymphocytes pathway’ was largely downregulated, with genes including CD70 (-5 fold), MAP3K9 (-7 fold) and tumour necrosis factor (TNF, 3.3 fold). CD8a also downregulated in pigs with CS pre-infestation. Week 0 also showed the downregulation of various pro-inflammatory response related molecules in CS including IL17F (-4.3 fold), and Th1 differentiation related STAT4 (-4.1 fold).

### Early (pre-clinical) gene expression profiles

At 1 and 2 wpi a number of genes generally produced during inflammation were down regulated in CS. Immunity related canonical pathways predicted to be inhibited in CS at 1 wpi included PPAR/RXR activation, STAT3 signalling, and NF-κB signalling (Table 3). Diseases, disorders and function networks associated with the 1 wpi and 2 wpi gene lists included Organismal Injury and Abnormalities (563 genes, p = 2.05E-04–2.76E-18), Molecular Transport (203 genes, p = 4.62E-05–4.90E-12) and Organismal Development (236 genes, p = 3.63E-05–1.71E.17). In contrast to the profile at week 0, acute phase signalling pathways were downregulated in CS at 1 and 2 wpi, with associated genes including Albumin (ALB, -4.3 fold), Interleukin 1B (IL1B -6 fold) and Serum Amyloid A (SAA1, -3 fold, only in CS). At 2 wpi, downregulated acute phase molecules included Ceruloplasmin (CP, -8.7 fold), C-reactive protein (CRP, -2.3 fold), Haptoglobin (HP, -3.1 fold) and IL6R (-3.4 fold).

**Fig 4:**
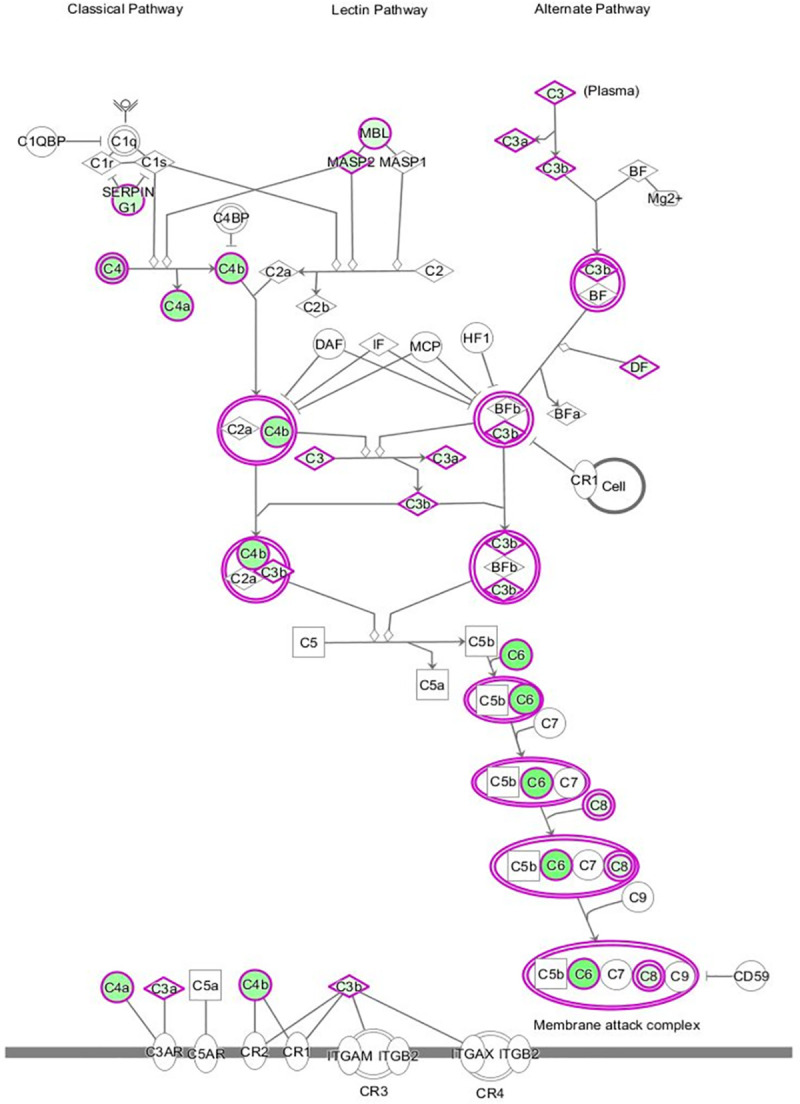
IPA canonical pathway depicting relationships among genes associated with Complement System at week 2 post-infestation in CS versus OS pigs. The differentially expressed genes included are those which showed ≥ ± 2 fold change in expression with p < 0.05. Individual nodes represent protein functions with relationships represented by edges. Nodes coloured by gene expression with green down- regulated genes and white indicating not differentially expressed genes.

Complement component C3 was downregulated in CS at 1 and 2 wpi (-2.1 and -2.4 fold), and C4B (-4.5 fold), C8G (-2.9 fold), complement factor D (CFD) and mannose binding lectin 2 (MBL2, -3 fold) were also downregulated at 2 wpi (Fig 4). Conversely, C6 was upregulated in CS (5 fold) at 1 wpi but downregulated at 2 wpi (-9.1 fold). Oncostatin M (OSM), a potent pro-inflammatory cytokine associated with various cutaneous inflammatory and allergic diseases, was downregulated (-6.7 fold) [23]. Venn diagram comparative analysis revealed that certain inflammatory related cytokines such as interleukin 8 (IL-8, -7.6 fold) and IL-19 (-6.4 fold) were only downregulated in CS in pre-clinical infestation. Despite the trend of most inflammatory genes being downregulated in early infestation, exceptions of slightly upregulated inflammation associated genes present only in CS included IL18 (2.1 fold). IL27, which can play both proinflammatory and anti-inflammatory roles, was exclusively upregulated at 1 wpi in CS (3.6 fold), but downregulated at 2 wpi relative to OS (-5.9 fold).

While some Toll like receptors (TLRs) were downregulated at 1 wpi (TLR3, -2 fold, TLR9, -3 fold, only in CS), TLR8 was upregulated by 44 fold. At 2wpi, TLR2 and TLR5 were also downregulated in CS (-4 and -3 fold respectively). MYD88 continued to be strongly downregulated only in CS (-9.7 fold) at 1 wpi, but was upregulated at 2 wpi (3.9 fold). Dendritic cell maturation and antigen presentation related genes were downregulated, including CD1B (-8 fold), Major histocompatibility complex (MHC) II (HLA-DOB,-2.8 fold, only in CS) MHC IA5 (HLA-5, -6 fold) and interleukin 12B and its receptor (-2 and -3 fold respectively, at 2 wpi). In OS a different pattern was evident, with Langerhans cell (CD207, 4 fold) and antigen presentation genes upregulated at 1 wpi (HLA-DOB, 3 fold, HLA-DQA1, 3 fold).

At 1 wpi, we detected downregulation of IL15 (-2.7 fold), IL19 (-7.5 fold, only in CS) and IL33 (-3.1 fold). These molecules are involved in leukocyte migration, secretion of pro-inflammatory mediators, proliferation of T-lymphocytes and apoptosis [24]. Downregulated immune cell recruitment and trafficking molecules at 2 wpi included CCL5 (-2 fold), Selectin P ligand (SELPLG, -4.1 fold), and intercellular adhesion molecule 3 (ICAM3, -6.2 fold).

As per week 0, the CD27 signalling pathway was inhibited in CS pigs at 1 wpi, with molecules including CD70 (-8 fold, exclusively in CS), MAP2K1 (-9.5 fold) and NFKB2 (-2.6 fold). While CD3G was strongly upregulated in CS (20 fold) and downregulated in OS (-6 fold) at 1 wpi, other T cell markers were downregulated in CS, including CD8A (-3 fold, only in CS), CD247 (-3 fold) and CD4 (-2 fold). Lymphocyte antigen 9 (LY9), an immunomodulatory receptor promoting Th17 differentiation was downregulated only in OS (-3 fold). Th1 pathways were predicted to be inhibited in CS at 2 wpi (Z score -2.8, 6.49E-0.3). STAT4 was upregulated in CS at 2 wpi (2.6 fold). At 1 and 2 wpi, transcription of certain immunoregulatory mediators was downregulated in CS, including IL27 (-5.9 fold) and transforming growth factor (TGF) β1 (-4.3 fold). Conversely TGFB (3 fold) and its negative modulator CD109 (3 fold) were upregulated in OS at 1 wpi. The immune-inhibitory CD274 (PDL1) gene expression was decreased only in CS skin at 1 wpi (-5 fold) but increased at 2 wpi (4 fold). Venn diagram comparative analysis comparing CS vs controls and OS vs Control at 1 wpi revealed that DCT (-69 fold) and adiponectin (ADIPOQ, -30 fold) were specifically downregulated in CS, congruent with observations at week 0. Other melanocyte pigmentation signalling pathways were widely inhibited, although curiously DCT was upregulated in CS at 2 wpi (18 fold), in contrast to other time points.

In summary, in preclinical CS infestation, we detected downregulation of Th1, innate immune and inflammatory responses, and antigen presentation indicating the presence of transcriptional control mechanisms to suppress Th1 biased and innate immune responses, and delayed antigen presentation early in the infestation in pigs with CS. There was early evidence to suggest promotion of Th2 and Th17 responses in CS. Conversely, in OS, antigen presentation genes were upregulated in early infestation.

### Later (clinical) gene expression profiles

**Fig 5:**
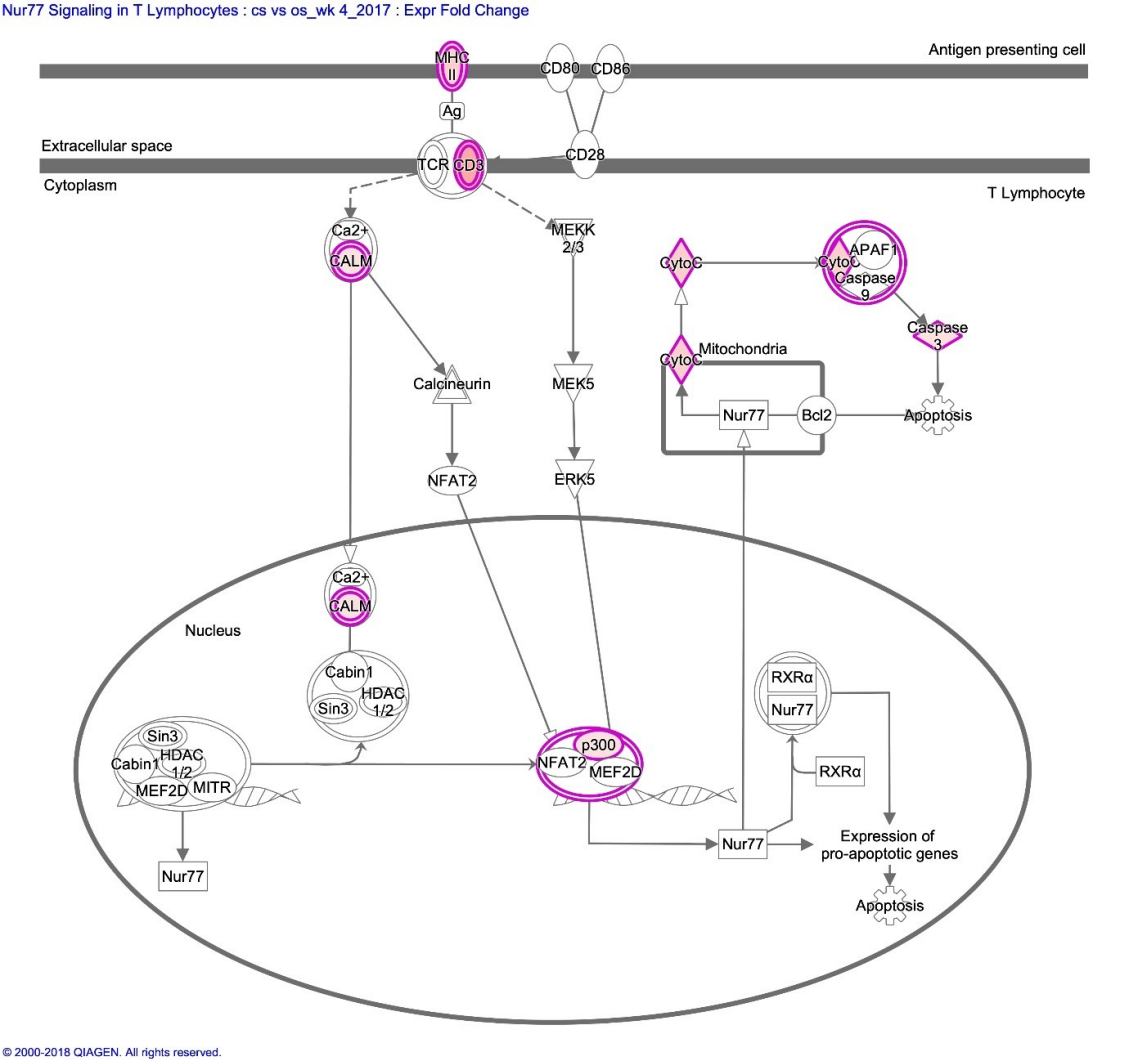
IPA canonical pathway depicting Nur77 signalling pathway in lymphocytes at week 4 post-infestation in CS versus OS pigs. The differentially expressed genes included are those which showed ≥ ± 2 fold change in expression with p < 0.05. Individual nodes represent protein functions with relationships represented by edges. Nodes coloured by gene expression with pink indicate up- regulated genes.

At 4 wpi, clinical signs of scabies first became evident (Fig 1). This appeared to correlate with a dramatic change in the trends of gene expression, compared to other time points, with a much lower proportion of DEGs, most of which were upregulated in CS vs OS. This changing profile may reflect a mix of mite-induced anti-inflammatory responses together with increasing skin damage and allergic responses. Significant canonical pathways included Nur77 signalling in T lymphocytes and Granzyme B signalling (Table 3, Fig 5). The T cell receptor protein CD3G (9 fold) and the major histocompatibility complex gene, MHC IA (HLA-A) (5 fold) are both involved in the Nur77 signalling pathway which promotes activation of T lymphocytes by antigen presenting cells. This pathway also results in apoptosis via Caspase 3 (CASP3, 2.2 fold) and somatic Cytochrome C (CYCS, 4.5-fold), and has been implicated in the inhibition of pro-inflammatory responses through the blocking of NF-kB signalling [25]. Additional DEGs of relevance in CS at 4 wpi, include STAT1 (3.8 fold), and the MHC II transcriptional activator Regulatory Factor X5 (RFX5, 11 fold). In addition, as observed at 1 wpi, TLR8 was again upregulated but to a lesser extent (2.8 fold). Expression of Fc fragment of IgG IIb (FCFR2B) was also increased (2 fold), along with LY96 (4 fold, also increased in CS pre-infestation).

**Fig 6:**
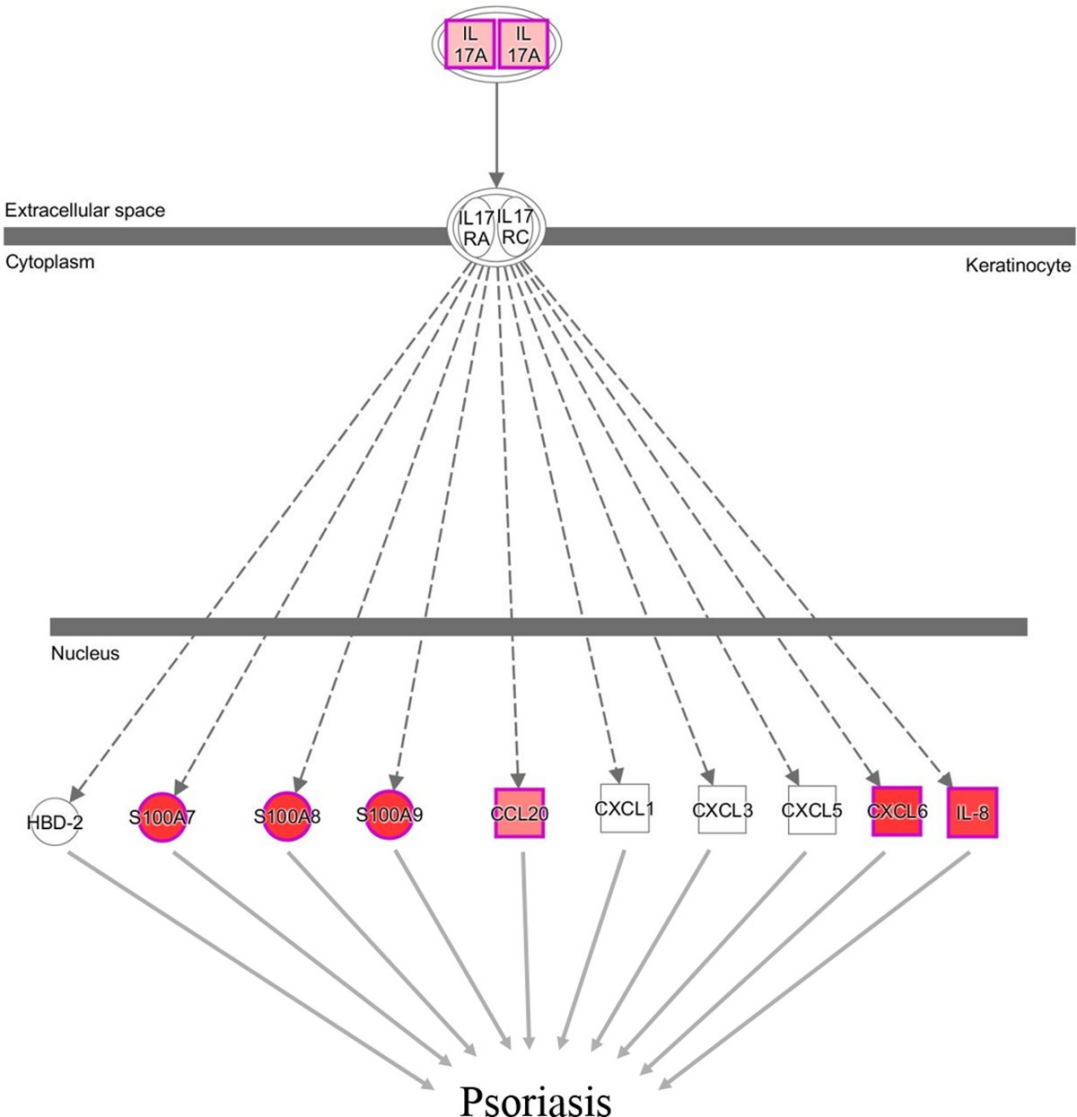
IPA canonical pathway depicting relationships among genes associated with Role of IL-17A and S100s –calcium binding proteins in Psoriasis at week 8 post infestation in CS vs OS pigs. The differentially expressed genes included are those which showed ≥ ± 2 fold change in expression with p < 0.05. Nodes coloured by gene expression with red (higher levels of expression) and pink nodes representing up regulated genes and white indicating not differentially expressed genes.

At 8 wpi, clinical phenotypes of CS or OS were readily discernable (Fig 1). In contrast to the profiles observed at 1 and 2 wpi, later CS infestation was associated with the upregulation of genes involved in inflammatory responses, antigen presentation and cell trafficking. Notable pathways included, “Role of IL-17A in Psoriasis” (Fig 6), “T Cell Receptor Signalling”, “CD28 Signalling in T helper Cells” and “IL-8 Signalling” (Table 3). Diseases, disorders and function networks associated with the gene list at 8 wpi included–Inflammatory Diseases (139 genes, p-value 1.17E-08–7.67E-21), Cell to Cell Signalling and Interaction (150 genes, p-value 3.16E-07–1.93E-28), Tissue Morphology (170 genes, p-value 3.12E-07–2.09E-24)”.

A number of canonical pathways were related to IL-17A mediated inflammatory processes in CS, with upregulation of IL17A (4.7 fold), S100 calcium binding proteins S100A7 (psoriasin 1, 21.5 fold), S100A8 (calgranulin A, 19.9 fold) and S100A9 (calgranulin B, 21.1 fold); CCL20 (9.1 fold), CXCL6 (64.32 fold) and IL8 (13.9 fold). Venn analysis comparing CS vs control and OS vs control samples indicated that several of these, and other IL-17A associated genes were exclusively upregulated in CS, including IL8, CXCL6, CCL20, IL17RB, CCL27, OSM, IL19 and IL-20. Conversely, IL17F was only upregulated in OS, and OSM was downregulated in OS at 8 wpi. Moreover, we observed strong upregulation of Arginase 1 (30.8 fold) and Arginase 2 (4 fold), which have been found to be associated with high IL-17A expression. Genes associated with antigen presentation and immune cell trafficking in CS were evident at 8 wpi, with HLA-3 being upregulated (2.6 fold), whereas this gene was exclusively upregulated at 1 wpi in OS. Chemokines CCL3L1 (2.1 fold, CCL17 (2.4 fold), CXCL2 (6.1 fold), selectin SELPLG (3.9 fold) and ICAM3 (3.3 fold) were all upregulated in CS at 8 wpi, with roles in immune cell activation and trafficking to sites of inflammation, and instigation and maintenance of the inflammatory responses. CXCL11, chemotactic for activated T cells, particularly in the skin, and macrophage associated CXCL16, were both downregulated in CS at 8 wpi. Immunosuppressive and Treg chemoattractive factor CCL4 was upregulated (2.4 fold) at 8 wpi in CS pigs. As observed at previous time points, CD3 markers were upregulated (CD3E, 3.4 fold, CD3G, 3.6 fold). Other differentially expressed cell surface markers at this time point included CD40LG (3.6 fold), which is involved in immunoglobulin class switching and IgE production. Conversely, the antigen presenting cell associated marker CD86 was downregulated in CS (-3.3 fold), and exclusively upregulated in OS vs control at 8 wpi. As well as the strong IL-17A response mentioned previously, an association of Th2 type cytokines at 8 wpi of CS infestation was evident, including IL-13 (2.4 fold) and IL-5 (exclusively upregulated in CS vs control, 3 fold). TGF beta, which promotes either T-helper 17 cells (Th17) or regulatory T-cells (Treg) lineage differentiation, was upregulated (2.2 fold). The melanin synthesis associated gene DCT continued to be downregulated in CS at 8 wpi (-6.4 fold), with this gene upregulated in OS vs control (4 fold) at this time point. Other genes showing downregulation late in the infestation included C3 (-2.6 fold), granulocyte colony-stimulating factor (CSF3, -2.8 fold) and its receptor CSF3R (-2.7 fold).

Collectively, the profile of CS vs OS at 8 wpi was one of a Th17 and neutrophil mediated inflammatory response, with chemotactic factors for T cells and Th2 related molecules.

### qRT-PCR validation of microarray data

To confirm the validity of the microarray results, eight DEGs identified in the 2-way ANOVA with a p-value of ≤ 0.05 and FC of > 2.0 were selected randomly and evaluated by qRT-PCR. qPCR assays were performed on the cDNA prepared from the same total RNA samples used for the microarray study. qPCR to validate mRNA expression of IFNγ, IL1β, FOXP3, glyoxalase I (GLO1), TGFβ, CD274, NLRP3 and TNF in skin derived from mite infested (OS, n = 4 and CS, n = 4) and non-infested control (C, n = 4) pigs revealed that the expression was confirmed for IFNγ, IL1β, FOXP3, GLO1, TGFβ, CD274, NLRP3 and TNF with >3.0 fold higher expression (S3 Table). These selected genes represented a range of genes expressed (upregulated) at different time points of the study period. qPCR expression of these selected genes over the time course of the infestation was considered to be validated as the fold change measured by both qPCR and microarray were > 2 fold.

## Discussion

This is the first comprehensive analysis comparing gene expression profiles in CS and OS, making use of a unique *in vivo* resource to identify significant differences in gene expression between the two clinical phenotypes. In general, CS was associated with initial delays in inflammatory responses, antigen presentation, immune cell trafficking and T cell proliferation, followed by strong upregulation of inflammatory and Th17 associated gene signatures in later infestation.

### Pre-infestation profiles and potential markers for susceptibility

One of the aims of this study was to investigate factors involved in underlying susceptibility to CS and as such, we compared gene expression profiles in pigs prior to infestation. Previous immune profiling of these same pigs showed no significant differences in CD4+, CD8+ or γδ+ T cell positive cells between groups at baseline [11]. However, principal component analysis in the present study demonstrated that pigs that eventually developed CS grouped separately from the OS pigs even at week 0, with many genes differentially expressed at this time point. Notwithstanding the potential lack of statistical power of these observations (see limitations), it is acknowledged that 3–4 weeks of age represents a “critical window” in immune development in piglets. High variation in immune parameters is expected at this time, particularly in outbred pigs, as used here [26, 27]. Indeed, early studies during the establishment of this porcine model showed that early age was necessary to facilitate successful mite infestation [13]. Additionally, all piglets at this time point were subjected to several stressors including early weaning, movement to the experimental facility and physical handling. Enhanced pro-inflammatory and acute phase responses observed in the CS group at week 0 may be indicative of a heightened susceptibility to stress in individual pigs, subsequently predisposing them to more severe infestation upon mite challenge [28]. Pigs that went on to develop CS also had transcriptional profiles at week 0 suggestive of potential dysregulation in macrophage associated pathways. Observations of no or very few macrophages have been noted previously in biopsies collected from CS patients [1]. The downregulation of MYD88, a key regulator of innate and adaptive immunity, is notable, as is suppression of CD27/70 signalling, suggesting possible impaired T-cell proliferation in CS pigs. In addition, IL-17F was downregulated in pigs that later developed CS, indicative of baseline differences in Th17 differentiation pathways between clinical phenotypes.

*S*. *scabiei*, like other parasites, exerts immunomodulatory effects on the host to facilitate successful infestation, and this is thought to explain the delayed appearance of clinical symptoms during a primary infestation. In a recent microarray analysis [29] investigators demonstrated that live scabies mites influence the expression of numerous genes in keratinocytes and fibroblasts in human skin equivalents (HSEs). Our results suggest that in CS, downregulation of immune and inflammatory response genes in early infestation was even more prominent, with many genes being exclusively downregulated in CS compared to OS and C pigs. Indeed, the obsevations of dramatic alterations in the numbers and trends of differentially expressed genes at week 4 compared to earlier and later weeks, in association with the first appearance of clinical signs suggests that this time point represented a critical “switch” between an immunomodulatory and strong inflammatory respose. Future studies should focus on further elucidating changes in host responses at this time point. In contrast, in OS skin, many genes were exclusively upregulated in early infestation. These upregulated genes may limit further mite proliferation as the immune response becomes activated, and could be the reason for low mite burden observed in OS at clinical presentation. In addition, upregulation of immunoregulatory molecules in OS pigs early in the infestation may suppress the pathogenic inflammatory T cells which contribute to skin pathology in CS.

### Decreased pathogen recognition and inflammatory signalling in early CS

Toll-like receptors (TLRs) play a fundamental role in pathogen recognition and activation of innate immune responses, and mediate the production of cytokines necessary for the downstream activation of effective cell-mediated immunity [30]. Several TLRs and associated TLR signalling pathway genes were downregulated early in CS infestation. TLR2 was differentially expressed at several time points, and most highly downregulated in CS at 2wpi. TLR2 mediates the innate immune response to bacterial pathogens and induces Th1 cytokine secretion [31] which may be suppressed in CS [1, 8]. LY96 (MD-2), upregulated at 1 and 2 wpi in OS, and 4wpi in CS, is a TLR4 accessory protein, involved in the promotion of inflammatory responses to LPS via myeloid differentiation primary response 88 (MYD88) [32]. One of the major allergens from the closely related house dust mite, Der p 2, has been shown to act as a functional mimic of MD-2, facilitating the activation of a pro-inflammatory response in the airway epithelium [33, 34]. LY96 enhances TLR2, TLR4 and the NF-κB signal transduction pathway [35]. A recent study showed that *Demodex* mite extracts decreased TLR2 expression in cultured sebocytes [36], although this may be mite density dependent, as higher mite numbers appear to be associated with activation of TLR inflammatory pathways and rosacea pathogenesis. Factors affecting this “switch” from TLR mediated immune modulation to stimulation are not clear [37]. In contrast, TLR8 was significantly upregulated in CS pigs at 1wpi. TLR8 signalling has been shown to play a role in reversing the suppressive function of Tregs [38] and accordingly TLR8 gene polymorphisms have been implicated in the susceptibility to autoimmune inflammation, allergic disorders, and increased IgE responses [39, 40] which are all characteristics of CS [1, 8].

Scabies-mite mediated inhibition of host complement has been previously reported [41]. Here, we show that while inhibition of complement pathways occurs at a transcriptional level in both clinical phenotypes, it was most pronounced in CS. Low levels of serum C3 and C4 have been noted in CS [5]. Low expression or the absence of C4 protein coincides with disease severity of systemic lupus erythematosus (SLE) and is the strongest genetic risk factor for SLE or lupus-like disease [42]. JAK/STAT pathways were also differentially expressed at multiple time points, indicating dysregulation in inflammatory signalling events. Enrichment of JAK/STAT signalling pathways has similarly been noted in *S*. *scabiei* infested rabbits [43]. JAK2 was downregulated at 1 and 2wpi in CS, and pathway analysis showed inhibition of STAT3 signalling in CS at both 1 and 2wpi. Importantly, STAT3 gene mutations have been associated with immunodeficiency, autoimmunity, recurring bacterial infections of the skin, increased circulating immunoglobulin E (IgE) and severe eczematoid rash [44, 45]. STAT3 gene variations are also associated with increased predisposition to psoriasis [44].

The NF-κB signal transduction pathway is well known for its role in inflammation, immunity, cell differentiation and apoptosis [46]. NF-κB regulated genes have been shown to influence the development of a rapid cutaneous inflammatory response to infestation with the ectoparasitic mite *Psoroptes ovis* in sheep, [47]. In contrast, we saw downregulation of NF-κB2 in CS pigs throughout infestation. Defects in this gene have been associated with primary immunodeficiency and autoimmunity. In addition, several other genes associated with the NF-κB signalling pathway were downregulated early in the infestation (1 and 2 wpi). This downregulation of NF-κB signalling, may potentially affect downstream T-cell and NK-cell activity, and impair innate and adaptive immune responses.

### Temporal changes in antigen presentation and immune cell trafficking in CS

A feature of this analysis has been evidence of delayed antigen presentation in CS, likely hindering the ability of pigs with CS to mount an effective adaptive immune response. Langerhans cells have been shown to increase in the skin of dogs with sarcoptic mange [48], but differences in dendritic cells between CS and OS have not been investigated until now. Our results showed that dendritic cell maturation pathway associated genes were significantly downregulated in CS at 1wpi. CD1B and CD1E were exclusively downregulated in CS at 1wpi but upregulated at 4 and 8wpi. Similarly, CD207 (langerin) was upregulated at 1wpi in OS, but at 8wpi in CS. CD86, expressed on antigen presenting cells and essential for T cell activation, was downregulated in CS at 2 and 8wpi. In addition, delayed transcription of MHC associated genes was observed in CS. Several of these antigen presentation genes were exclusively upregulated in CS at 8wpi, including HLA-DRA, HLA-DMB, HLA-DQA1 and HLA-DQB1. At this later time point, the higher mite numbers in CS would be expected to provide higher levels of antigenic stimuli leading to increased MHC expression and strong host humoral and cell mediated responses observed at this time of infestation in CS.

Selectins and chemokines have been implicated in various inflammatory skin diseases. Selectin P ligand (SELPLG) was downregulated in CS at 2 wpi but upregulated at 8wpi. Conversely, Selectin P (SELP) was upregulated in OS relative to control at 2 wpi. SELPLG encodes a glycoprotein mediating leukocyte trafficking during the initial stages of inflammation. Aberrant SELPLG expression results in defective innate and adaptive immune responses [49]. Cutaneous T cell-attracting chemokine (CTACK/CCL27) is predominantly expressed in the skin by keratinocytes during the local immune response [50] and is chemotactic for skin associated memory T cells. Scabies mite extracts have been shown to induce CCL27 in human skin equivalents [51]. Notably, CCL27 and its receptor, CCR10 were upregulated only in OS at 1 wpi. CCL27-CCR10 interactions are thought to play a key role in T cell-mediated skin inflammation [50].

We also detected exclusive upregulation of chemokines involved in neutrophil chemotaxis in CS at 8 wpi, most notably 64-fold upregulation of CXCL6 (also known as granulocyte chemotactic protein 2, GCP-2). CXCL6 complements the activity of IL8 as a neutrophil chemoattractant and activator, so this concords with the upregulation of IL8 in CS. Overall, downregulation of chemokines and selectins early in CS might inhibit trafficking of immune effector cells to the skin. This early suppression may also impair local tissue immunity allowing mites to increase in number, promoting the progress of the disease and the clinical outcomes seen at later stages of the disease.

### Immune modulation in early CS infestation facilitates mite proliferation

We found differential transcription of several genes involved in immune regulation throughout the time course of *S*. *scabiei* infestation. Transcription of TGFβ1 was downregulated at 1 wpi, and upregulated at 8 wpi in pigs with CS. Levels of TGFβ influence Treg/Th17 differentiation, and its early suppression in CS may promote later Th17 responses. FOXP3 is essential for the development and immunosuppressive function of Treg cells [52], and *P*. *ovis* mite infestation in sheep has been shown to result in the infiltration of Foxp3 ^(+)^ T cells into the skin [53]. It has been previously demonstrated that *S*. *scabiei* extracts stimulate T regulatory (Treg) cells to produce immunosuppressive cytokines in PBMCs [54]. Dysregulation of FOXP3 in CS was observed at several time points, being upregulated at 1 and 8 wpi, but downregulated at 2 and 4 wpi. While further investigation is needed, it is possible that ongoing dysregulation may promote Th17 proliferation and the uncontrolled inflammatory responses observed in later weeks. In a similar trend, IL-27 was initially upregulated in CS at 1 wpi, but then downregulated at 2 wpi. IL-27 is a potent inhibitor of Th2 and Th17 cell development in helminth [55] and protozoan [56] infection, hence its downregulation may promote the amplified Th2 and Th17 responses observed in later CS infestation. Additionally, we observed dysregulation of the immunomodulatory ligand CD274 (PD-L1). This gene was downregulated at 1 wpi in CS, switching to upregulated at 2 wpi. This ligand blocks T cell activation and plays a key role in tumor development and autoimmunity. Decreased expression has been associated with active episodes of SLE [57]. Our results suggest that the early dysregulation of these immunomodulatory genes in the skin may lead to the strong inflammatory milieu of CS observed in later infestation.

### Alterations to cellular immune profiles in crusted scabies

As previously mentioned, CD70 and the “CD27 Signalling in Lymphocytes” pathway were downregulated in early CS. Upon engagement with CD27 on antigen activated T cells, CD70 signals DCs to promote T cell proliferation [58]. CD27-CD70 interactions provide key contributions in promoting effector and memory CD8^+^ T cell formation and survival enhancing CD8^+^ T responses [59]. This is supported by the decrease in CD8A at 1 wpi. Conversely, ICOS, FADD and MAL were exclusively upregulated in OS relative to control at 1wpi. These molecules are of importance to T-cell signal transduction, activation and proliferation [60, 61]. While the above suggests that early effector T cell responses may be diminished in CS, CD3G was markedly upregulated at 2, 4 and 8 wpi. This could potentially be related to the increase in gamma delta T cells, which are known to increase from 1 wpi in CS [11]. At 8 wpi CD8A and Granzyme B (GZMB) were upregulated, consistent with observations of increased CD8+ cells in the skin of CS.

Th2 biased and allergic responses have been implicated in the pathogenesis of various inflammatory skin diseases such as psoriasis, SLE, and atopic dermatitis. Our results were consistent with our previous observations, with increased expression of IL4, IL5 and IL13 in CS at 8 wpi, with IL5 and IL13 exclusively upregulated in CS. Additionally, we observed CD40L upregulation at 8 wpi in CS pigs. CD40L, upon cross-linking with CD40 generates a costimulatory signal which results in T and B cell proliferation, antibody isotype switching in B cells and enhances production of IL-4 and IgE [62]. IL17RB was upregulated in CS. This receptor is specific for the proinflammatory cytokines IL-17B and IL-17E. IL-17E amplifies Th2 immune responses by inducing Th2 cytokine secretion and is also able to induce IgE production and eosinophilia [63].

### Skin pathology in crusted scabies is associated with strong Th17 inflammatory responses

Recent studies by our group demonstrated striking upregulation of IL-17 secreting T cells in the skin of pigs with CS [11, 12]. Similarly, IL-17 is associated with severity of *P*. *ovis* infestation in susceptible Belgian Blue cattle [64]. Results from the present study provide further evidence for Th17 type involvement in CS, with polarization to these responses apparent from very early infestation, well before the appearance of clinical signs. At 8 wpi exclusive expression of powerful inflammatory mediators including IL17A, CXCL6 and CCL20 was observed in CS compared to OS skin samples. We also detected upregulation of IL8 and its receptor IL8R. These molecules have been implicated in pathogenesis of psoriasis and contribute to the psoriatic condition by increasing the migration of effector immune cells to the sites of inflammation [65].

The longitudinal approach of this analysis provided opportunities to explore factors leading to the strong IL-17 inflammatory response observed in later infestation. As previously mentioned, the potential dysregulation of IL27 may promote Th2 and Th17 polarization. Additionally, we found early downregulation of CD5L in CS skin. CD5L affects the expression of pro-inflammatory genes and its upregulation inhibits the pathogenic function of Th17 cells [66, 67], suggesting that its downregulation in CS might be another mechanism of promoting the activities of IL17 producing cells. Additionally, Arginase 1 (ARG1) was among the DEGs with highest fold change at 8 wpi. Increased arginase activity in peripheral blood has been demonstrated during murine and human *Schistosoma mansoni* infection [68, 69]. Arginase has been shown in peripheral blood of patients with active SLE, with levels correlating with disease severity [70]. In these patients Arginase is produced by Myeloid-derived suppressor cells (MDSCs), which are highly efficient in stimulating Th17 cell differentiation and promoting tissue inflammation [70]. Furthermore, MDSCs from SLE patients displayed increased capacity to drive Th17 cell differentiation in an Arginase 1-dependent manner [70]. Previously, ARG1 was also found to be induced by scabies mites in human skin equivalents [29]. Our results indicate that ARG1 production may further exacerbate inflammation by promoting Th17 cell differentiation and IL-17 production in CS.

IL19 was downregulated early, and then upregulated late in infestation. IL-19 is produced under inflammatory conditions, inducing Th2 cytokines from activated T cells. IL-19 might play an important role in the pathogenesis of asthma [71]. Additionally, IL-19 is linked to both Th2 and Th17 responses, with increased IL19 expression reported in skin lesions of psoriatic patients and elevated IL-19 serum levels correlated with psoriasis severity. IL-19 is induced in keratinocytes by IL-17A and has been shown to amplify the production of S100A7/8/9, IL-1β and IL-20 [72]. IL-20, is a proinflammatory cytokine produced by keratinocytes, monocytes and endothelial cells, and was observed exclusively upregulated at 8 wpi in CS skin. Scabies mites have been previously reported to induce IL-20 in human skin equivalents [29]. During inflammation IL-20 mediates keratinocyte proliferation and is implicated in epidermal hyperplasia [73]. Therefore, upregulation of IL20, may play a role in keratinocyte proliferation and the development of thickened epidermis, scales and crusts observed in CS.

The S100 calcium signalling proteins were among the most highly differentially expressed genes in our data, particularly at 8 wpi. These proteins are important mediators of acute and chronic inflammatory diseases such as psoriasis, AD, arthritis, atherosclerosis and microbial infections [74]. Psoriasin (S100A7) is an important effector molecule of the cutaneous barrier and is abundantly expressed in psoriatic and AD keratinocytes with epidermal barrier disruption significantly enhancing psoriasin expression [75, 76]. Calgranulins S100A8 and S100A9 are markers of keratinocyte activation and have been demonstrated to play a role in hyper-proliferation and abnormal differentiation of keratinocytes in psoriasis [77] and also result in the development of severe crusted lesions in sheep scab [78]. IL-17A promotes S1000A7, S100A8 and S1000A9 expression [76]. This increased expression of S100 genes in mite infested skin could further amplify an already prolific inflammatory response and our results also indicate their role in the development of skin lesions and crusts observed in CS cases.

Another consistently highly differentially expressed gene was Dopachrome Tautomerase (DCT), associated with the synthesis of melanin. DCT was strongly downregulated at 1 wpi (exclusively in CS) and upregulated at 2 wpi, and then downregulated again moderately at 8 wpi. Interestingly, decreased DCT expression has been detected in skin lesions from patients with Vitiligo [79]. Hence, dysregulated DCT expression may be associated with disrupted melanin synthesis in CS, partially explaining the widespread depigmentation of skin often observed in this disease.

### Limitations

For differential gene expression, we applied a FC cut-off of ≥ 2 to reduce false positives, with the assumption that larger FC cut-offs will preferentially select genes with the most biological significance. Genes with a smaller degree of differential expression (e.g., IL22 and IL23) observed in other inflammatory skin diseases [80, 81] may have not passed this FC cut-off, potentially preventing the identification of significant disease related pathways and associations with host response. Importantly, it is acknowledged that the study may be underpowered, as only one skin sample per animal at each time point was examined. While lack of biological replicates is a known source of variation in transcriptomic studies, the numbers of biopsies collected from the pig ears was limited by ethical considerations, being mindful that additional biopsies were also collected for cellular immunology and histology studies in the overall work related to this trial. The sample size used in this study is similar to other transcriptomic studies of human and animal parasitic infestations, noting the difficulties in obtaining sufficient samples with clearly defined clinical phenotypes. Samples sizes were selected in consulation with statisicians based on clinical phenotype data from lesion scores and pilot transcriptional studies by qPCR [12] from a number of previous trials, which suggested that despite small animal numbers, large effect sizes would be apparent and thus degree of statistical power adequate for a study of this nature.

### Conclusion and future directions

In summary, our transcriptomic analysis of lesional skin in CS relative to OS has revealed dysregulation of many genes associated with pathophysiologic pathways of autoimmune inflammatory disorders such as psoriasis, rheumatoid arthritis, AD, and SLE. It has provided key insights into the broader proinflammatory and allergic responses in CS and role of various immune effectors cells, molecules and associated pathways in the development of the CS phenotype. Importantly, this study has provided new insights into the early, pre-clinical molecular events likely promoting the strong Th2 and Th17 responses and severe skin pathology observed at clinical presentation in CS. For example, dysregulation of skin associated inflammatory chemokines such as CCL27 may play a role in the timing of initiation and enhancement of skin inflammation by mediating T cell recruitment to the skin lesions. Changes in the expression of molecules such as IL27, IL19, CD5L and ARG1 are all conducive to the proliferation of pathogenic Th17 cells, as is the possible altered Treg/Th17 effector balance in early infestation. As previously noted [11, 12], TLR8, IL-23 and IL-17 are promising immunotherapeutic targets and their antagonism might be a promising therapy for the treatment of CS in combination with acaricides. Immune based therapies are currently in clinical trials for various inflammatory diseases and efficacy has been seen in clinical trials in psoriasis [82, 83]. Arginase 1 and 2, induced in myeloid cells, MDSCs, macrophages and neutrophils, by pro-inflammatory cytokines, are among the main arginine-catabolizing enzymes involved in inflammatory immune responses during pathological conditions through mechanisms that use mediators of this unique metabolic pathway [84, 85]. Our results indicate that the strong upregulation of ARG1 may promote immune dysfunction by depriving T cells of essential metabolites such as arginine. The inflammation observed in CS pigs late in the infestation may cause expansion of MDSCs which may result in expression of ARG1 promoting Th17 cell proliferation and thereby enhance the inflammation and disease activity in CS. A better understanding of arginine metabolic pathways within the inflammatory environments in CS cases, and serological profiling of Arginase in CS vs OS would be of interest.

STAT1, STAT3, TLR8, IL13, RFX5 gene polymorphisms have been demonstrated as susceptibility loci for the immune dysregulation in various inflammatory and allergic diseases. In the future, these loci can be an area of investigation to ascertain the association of genetic risk factors in immune dysregulation and CS pathogenesis. In addition to this, genetic variants in STAT6, high affinity receptor for IgE (FCER1A) and HLA-DRB1 genes have been found to influence susceptibility to various infectious and immune-mediated diseases [86, 87], and could be potential determinants of impaired immune response and high IgE concentration in CS.

Although transcriptomic profiling can be highly informative at an overall level, genes encode proteins and it is the proteins that ultimately dictate various cellular functions. In this regard, our results provide an important basis for the design of future functional characterisation studies using protein expression and gene inhibitory assays to investigate the precise role of specific genes to gain insights into the genes of interest in scabies.

While this study has provided substantial insights into changes in transcriptional profiles related to different clinical manifestations of *Sarcoptes scabiei* infestation, it must be emphasized that these observations are based on porcine infestation and cannot be automatically extrapolated to human scabies. However, noting the utility and appropriateness of porcine models to diseases of human skin [88] our results now provide a strong platform for further investigation of the immunopathogenesis of scabies in humans. Finally, integration of transcriptome information, with epigenomic, proteomic and functional data will, in the near future, create novel opportunities to pinpoint vital processes underlying scabies disease susceptibility and pathogenesis and increase the potential to identify novel targets for this important neglected disease.

## Supporting information

S1 Fig Box and whisker profile plot to examine the feature intensity distributions of microarray data over the time course of infestation with *S*. *scabiei*. Each row represents a single sample.
(DOCX)

S2 Fig IPA canonical pathway depicting relationships among genes associated with the Acute Phase signalling pathway in CS vs OS prior to experimental infestation. The differentially expressed genes included are those which showed ≥ ± 2 fold change in expression with p < 0.05. Nodes coloured by gene expression with red (higher levels of expression) and pink nodes representing up regulated genes.
(DOCX)

S1 Table. Primer sequences and amplicon details for porcine qRT-PCR (DOCX)

S2 Table Differentially expressed genes in Crusted vs Ordinary scabies pigs upon infestation with *S*. *scabiei*. Genes that differed significantly in expression (either up or down regulated) levels upon mite infestation over the time course of 8 weeks. Gene expression was deduced by a 2- way ANOVA combined with a Fisher's Least Significant Difference (LSD) post-hoc test. These genes had a FDR corrected p-value ≤ 0.05 and fold change ≥ ± 2.0. The gene function was verified with online databases IPA, UniProt and GeneCards. The notable genes associated with signalling pathways and gene networks in the processes of immune, inflammatory and allergic responses are indicated.
(DOCX)

S3 Table Confirmation of eight upregulated genes in microarray data by qPCR. These genes were identified in the 2-way ANNOVA analysis with a p-value of ≤ 0.05 and FC of > 2.0. Relative quantification of gene expression levels was determined by normalising to the HPRT1 control using the comparative Ct method with the formula 2 ^-ΔΔCT^ and expressed as fold change. Duplicates of each cDNA sample from infested (OS, n = 4; CS, n = 4) and non-infested controls (C, n = 4) were maintained. Wpi = weeks post-infestation.
(DOCX)

## References

[1] Walton SF, Pizzutto S, Slender A, Viberg L, Holt D, Hales BJ, et al Increased allergic immune response to *Sarcoptes scabiei* antigens in crusted versus ordinary scabies. Clin Vaccine Immunol. 2010;17(9):1428–38. 10.1128/CVI.00195-10 20631334 PMC2944463

[2] Lokuge B, Kopczynski A, Woltmann A, Alvoen F, Connors C, Guyula T, et al Crusted scabies in remote Australia, a new way forward: lessons and outcomes from the East Arnhem Scabies Control Program. Med J Aust. 2014;200(11):644–8. 10.5694/mja14.00172 . 24938345

[3] Walton SF, Currie BJ, Kemp DJ. A DNA fingerprinting system for the ectoparasite *Sarcoptes scabiei*. Mol Biochem Parasitol. 1997;85(2):187–96. 10.1016/s0166-6851(96)02825-3 . 9106192

[4] Hay RJ, Steer AC, Engelman D, Walton S. Scabies in the developing world—its prevalence, complications, and management. Clin Microbiol Infect. 2012;18:313–23. 10.1111/j.1469-0691.2012.03798.x 22429456

[5] Roberts LJ, Huffam SE, Walton SF, Currie BJ. Crusted scabies: clinical and immunological findings in seventy-eight patients and a review of the literature. J Infect. 2005;50(5):375–81. 10.1016/j.jinf.2004.08.033 . 15907543

[6] Cassell JA, Middleton J, Nalabanda A, Lanza S, Head MG, Bostock J, et al Scabies outbreaks in ten care homes for elderly people: a prospective study of clinical features, epidemiology, and treatment outcomes. Lancet Infect Dis. 2018;18(8):894–902. 10.1016/S1473-3099(18)30347-5 ; 30068499 PMC6060176

[7] Mounsey KE, Murray HC, King M, Oprescu F. Retrospective analysis of institutional scabies outbreaks from 1984 to 2013: lessons learned and moving forward. Epidemiol Infect. 2016;144(11):2462–71. 10.1017/S0950268816000443 . 27019288 PMC9150521

[8] Bhat SA, Mounsey KE, Liu X, Walton SF. Host immune responses to the itch mite, *Sarcoptes scabiei*, in humans. Parasit Vectors. 2017;10(1):385 Epub 2017/08/12. 10.1186/s13071-017-2320-4 ; 28797273 PMC5553898

[9] Falk ES, Matre R. In situ characterization of cell infiltrates in the dermis of human scabies. Am J Dermatopathol. 1982;4(1):9–15. . 7044174

[10] Walton SF, Beroukas D, Roberts-Thomson P, Currie BJ. New insights into disease pathogenesis in crusted (Norwegian) scabies: the skin immune response in crusted scabies. Br J Dermatol. 2008;158(6):1247–55. 10.1111/j.1365-2133.2008.08541.x . 18422789

[11] Liu X, Walton SF, Murray HC, King M, Kelly A, Holt DC, et al Crusted scabies is associated with increased IL-17 secretion by skin T cells. Parasite Immunol. 2014;36(11):594–604. 10.1111/pim.12129 . 25040151

[12] Mounsey KE, Murray HC, Bielefeldt-Ohmann H, Pasay C, Holt DC, Currie BJ, et al Prospective study in a porcine model of *Sarcoptes scabiei* indicates the association of Th2 and Th17 pathways with the clinical severity of scabies. PLoS Negl Trop Dis. 2015;9(3):e0003498 10.1371/journal.pntd.0003498 ; 25730203 PMC4346266

[13] Mounsey K, Ho MF, Kelly A, Willis C, Pasay C, Kemp DJ, et al A tractable experimental model for study of human and animal scabies. PLoS Negl Trop Dis. 2010;4(7):e756 10.1371/journal.pntd.0000756 ; 20668508 PMC2907415

[14] Schroeder A, Mueller O, Stocker S, Salowsky R, Leiber M, Gassmann M, et al The RIN: an RNA integrity number for assigning integrity values to RNA measurements. BMC Mol Biol. 2006;7:3 10.1186/1471-2199-7-3 ; 16448564 PMC1413964

[15] Bolstad BM, Irizarry RA, Astrand M, Speed TP. A comparison of normalization methods for high density oligonucleotide array data based on variance and bias. Bioinformatics. 2003;19(2):185–93. 10.1093/bioinformatics/19.2.185 . 12538238

[16] Benjamini Y, Hochberg Y. Controlling the false discovery rate: a practical and powerful approach to multiple testing. Journal of the Royal Statistical Society Series B. 1995;57:289–300.

[17] Brazma A, Hingamp P, Quackenbush J, Sherlock G, Spellman P, Stoeckert C, et al Minimum information about a microarray experiment (MIAME)-toward standards for microarray data. Nat Genet. 2001;29(4):365–71. 10.1038/ng1201-365 . 11726920

[18] Levast B, de Monte M, Chevaleyre C, Melo S, Berri M, Mangin F, et al Ultra-early weaning in piglets results in low serum IgA concentration and IL17 mRNA expression. Vet Immunol Immunopathol. 2010;137(3–4):261–8. 10.1016/j.vetimm.2010.06.004 . 20591504

[19] Petrov A, Beer M, Blome S. Development and validation of a harmonized TaqMan-based triplex real-time RT-PCR protocol for the quantitative detection of normalized gene expression profiles of seven porcine cytokines. PLoS One. 2014;9(9):e108910 10.1371/journal.pone.0108910 ; 25268123 PMC4182501

[20] Nygard AB, Jorgensen CB, Cirera S, Fredholm M. Selection of reference genes for gene expression studies in pig tissues using SYBR green qPCR. BMC Mol Biol. 2007;8:67 10.1186/1471-2199-8-67 ; 17697375 PMC2000887

[21] Schmittgen TD, Livak KJ. Analyzing real-time PCR data by the comparative C(T) method. Nat Protoc. 2008;3(6):1101–8. 10.1038/nprot.2008.73 . 18546601

[22] Rampton M, Walton SF, Holt DC, Pasay C, Kelly A, Currie BJ, et al Antibody responses to *Sarcoptes scabiei* apolipoprotein in a porcine model: relevance to immunodiagnosis of recent infection. PLoS One. 2013;8(6):e65354 10.1371/journal.pone.0065354 ; 23762351 PMC3675102

[23] Boniface K, Diveu C, Morel F, Pedretti N, Froger J, Ravon E, et al Oncostatin M secreted by skin infiltrating T lymphocytes is a potent keratinocyte activator involved in skin inflammation. J Immunol. 2007;178(7):4615–22. 10.4049/jimmunol.178.7.4615 . 17372020

[24] Liao YC, Liang WG, Chen FW, Hsu JH, Yang JJ, Chang MS. IL-19 induces production of IL-6 and TNF-alpha and results in cell apoptosis through TNF-alpha. J Immunol. 2002;169(8):4288–97. 10.4049/jimmunol.169.8.4288 . 12370360

[25] Kurakula K, Vos M, Logiantara A, Roelofs JJ, Nieuwenhuis MA, Koppelman GH, et al Nuclear Receptor Nur77 Attenuates Airway Inflammation in Mice by Suppressing NF-kappaB Activity in Lung Epithelial Cells. J Immunol. 2015;195(4):1388–98. 10.4049/jimmunol.1401714 . 26170382

[26] Nguyen DN, Jiang P, Frokiaer H, Heegaard PM, Thymann T, Sangild PT. Delayed development of systemic immunity in preterm pigs as a model for preterm infants. Sci Rep. 2016;6:36816 10.1038/srep36816 ; 27830761 PMC5103294

[27] Butler JE, Sinkora M, Wertz N, Holtmeier W, Lemke CD. Development of the neonatal B and T cell repertoire in swine: implications for comparative and veterinary immunology. Vet Res. 2006;37(3):417–41. 10.1051/vetres:2006009 . 16611556

[28] Ceciliani F, Ceron JJ, Eckersall PD, Sauerwein H. Acute phase proteins in ruminants. J Proteomics. 2012;75(14):4207–31. 10.1016/j.jprot.2012.04.004 . 22521269

[29] Morgan MS, Arlian LG, Markey MP. *Sarcoptes scabiei* mites modulate gene expression in human skin equivalents. PLoS One. 2013;8(8):e71143 10.1371/journal.pone.0071143 ; 23940705 PMC3733868

[30] Pasare C, Medzhitov R. Toll-like receptors: linking innate and adaptive immunity. Microbes Infect. 2004;6(15):1382–7. 10.1016/j.micinf.2004.08.018 . 15596124

[31] Lancioni CL, Li Q, Thomas JJ, Ding X, Thiel B, Drage MG, et al *Mycobacterium tuberculosis* lipoproteins directly regulate human memory CD4(+) T cell activation via Toll-like receptors 1 and 2. Infect Immun. 2011;79(2):663–73. 10.1128/IAI.00806-10 ; 21078852 PMC3028837

[32] Song DH, Lee JO. Sensing of microbial molecular patterns by Toll-like receptors. Immunol Rev. 2012;250(1):216–29. 10.1111/j.1600-065X.2012.01167.x . 23046132

[33] Tsai JJ, Liu SH, Yin SC, Yang CN, Hsu HS, Chen WB, et al Mite allergen Der-p2 triggers human B lymphocyte activation and Toll-like receptor-4 induction. PLoS One. 2011;6(9):e23249 10.1371/journal.pone.0023249 ; 21909400 PMC3167811

[34] Trompette A, Divanovic S, Visintin A, Blanchard C, Hegde RS, Madan R, et al Allergenicity resulting from functional mimicry of a Toll-like receptor complex protein. Nature. 2009;457(7229):585–8. 10.1038/nature07548 19060881 PMC2843411

[35] Dziarski R, Wang Q, Miyake K, Kirschning CJ, Gupta D. MD-2 enables Toll-like receptor 2 (TLR2)-mediated responses to lipopolysaccharide and enhances TLR2-mediated responses to Gram-positive and Gram-negative bacteria and their cell wall components. J Immunol. 2001;166(3):1938–44. 10.4049/jimmunol.166.3.1938 . 11160242

[36] Lacey N, Russell-Hallinan A, Zouboulis CC, Powell FC. Demodex mites modulate sebocyte immune reaction: possible role in the pathogenesis of rosacea. Br J Dermatol. 2018;179(2):420–30. 10.1111/bjd.16540 . 29532463

[37] Forton FMN. Elucidating the role of *Demodex folliculorum* in the pathogenesis of rosacea: exciting first steps. Br J Dermatol. 2018;179(2):252–3. 10.1111/bjd.16792 . 30024649

[38] Peng G, Guo Z, Kiniwa Y, Voo KS, Peng W, Fu T, et al Toll-like receptor 8-mediated reversal of CD4+ regulatory T cell function. Science. 2005;309(5739):1380–4. 10.1126/science.1113401 . 16123302

[39] Moller-Larsen S, Nyegaard M, Haagerup A, Vestbo J, Kruse TA, Borglum AD. Association analysis identifies TLR7 and TLR8 as novel risk genes in asthma and related disorders. Thorax. 2008;63(12):1064–9. 10.1136/thx.2007.094128 . 18682521

[40] Nilsson D, Andiappan AK, Hallden C, De Yun W, Sall T, Tim CF, et al Toll-like receptor gene polymorphisms are associated with allergic rhinitis: a case control study. BMC Med Genet. 2012;13:66 10.1186/1471-2350-13-66 22857391 PMC3459792

[41] Swe PM, Fischer K. A scabies mite serpin interferes with complement-mediated neutrophil functions and promotes staphylococcal growth. PLoS Negl Trop Dis. 2014;8(6):e2928 10.1371/journal.pntd.0002928 24945501 PMC4063749

[42] Yang Y, Chung EK, Zhou B, Lhotta K, Hebert LA, Birmingham DJ, et al The intricate role of complement component C4 in human systemic lupus erythematosus. Curr Dir Autoimmun. 2004;7:98–132. 10.1159/000075689 . 14719377

[43] He R, Gu X, Lai W, Peng X, Yang G. Transcriptome-microRNA analysis of *Sarcoptes scabiei* and host immune response. PLoS One. 2017;12(5):e0177733 10.1371/journal.pone.0177733 ; 28542251 PMC5441584

[44] Hillmer EJ, Zhang H, Li HS, Watowich SS. STAT3 signaling in immunity. Cytokine Growth Factor Rev. 2016;31:1–15. 10.1016/j.cytogfr.2016.05.001 27185365 PMC5050093

[45] Holland SM, DeLeo FR, Elloumi HZ, Hsu AP, Uzel G, Brodsky N, et al STAT3 mutations in the hyper-IgE syndrome. N Engl J Med. 2007;357(16):1608–19. 10.1056/NEJMoa073687 . 17881745

[46] Hayden MS, Ghosh S. NF-kappaB in immunobiology. Cell Res. 2011;21(2):223–44. 10.1038/cr.2011.13 21243012 PMC3193440

[47] Burgess ST, McNeilly TN, Watkins CA, Nisbet AJ, Huntley JF. Host transcription factors in the immediate pro-inflammatory response to the parasitic mite *Psoroptes ovis*. PLoS One. 2011;6(9):e24402 10.1371/journal.pone.0024402 21915322 PMC3168495

[48] Stemmer BL, Arlian LG, Morgan MS, Rapp CM, Moore PF. Characterization of antigen presenting cells and T-cells in progressing scabietic skin lesions. Vet Parasitol. 1996;67(3–4):247–58. 10.1016/s0304-4017(96)01038-2 . 9017872

[49] Somers WS, Tang J, Shaw GD, Camphausen RT. Insights into the molecular basis of leukocyte tethering and rolling revealed by structures of P- and E-selectin bound to SLe(X) and PSGL-1. Cell. 2000;103(3):467–79. 10.1016/s0092-8674(00)00138-0 . 11081633

[50] Xiong N, Fu Y, Hu S, Xia M, Yang J. CCR10 and its ligands in regulation of epithelial immunity and diseases. Protein Cell. 2012;3(8):571–80. 10.1007/s13238-012-2927-3 ; 22684736 PMC4430102

[51] Morgan MS, Arlian LG. Response of human skin equivalents to *Sarcoptes scabiei*. J Med Entomol. 2010;47(5):877–83. 10.1603/me10012 20939384 PMC2955294

[52] Sakaguchi S, Miyara M, Costantino CM, Hafler DA. FOXP3+ regulatory T cells in the human immune system. Nat Rev Immunol. 2010;10(7):490–500. 10.1038/nri2785 . 20559327

[53] McNeilly TN, McIntyre J, Frew D, Griffiths DJ, Wattegedera SR, van den Broek A, et al Infestation of sheep with *Psoroptes ovis*, the sheep scab mite, results in recruitment of Foxp3(+) T cells into the dermis. Parasite Immunol. 2010;32(5):361–9. 10.1111/j.1365-3024.2009.01196.x . 20500665

[54] Arlian LG, Morgan MS, Paul CC. Evidence that scabies mites (Acari: Sarcoptidae) influence production of interleukin-10 and the function of T-regulatory cells (Tr1) in humans. J Med Entomol. 2006;43(2):283–7. 10.1603/0022-2585(2006)043[0283:etsmas]2.0.co;2 . 16619612

[55] Anuradha R, Munisankar S, Bhootra Y, Dolla C, Kumaran P, Nutman TB, et al Modulation of CD4(+) and CD8(+) T Cell Function and Cytokine Responses in *Strongyloides stercoralis* Infection by Interleukin-27 (IL-27) and IL-37. Infect Immun. 2017;85(11). 10.1128/IAI.00500-17 28874444 PMC5649007

[56] Quirino GFS, Nascimento MSL, Davoli-Ferreira M, Sacramento LA, Lima MHF, Almeida RP, et al Interleukin-27 (IL-27) Mediates Susceptibility to Visceral Leishmaniasis by Suppressing the IL-17-Neutrophil Response. Infect Immun. 2016;84(8):2289–98. 10.1128/IAI.00283-16 27245409 PMC4962641

[57] Mozaffarian N, Wiedeman AE, Stevens AM. Active systemic lupus erythematosus is associated with failure of antigen-presenting cells to express programmed death ligand-1. Rheumatology (Oxford). 2008;47(9):1335–41. 10.1093/rheumatology/ken256 18650228 PMC2722808

[58] Borst J, Hendriks J, Xiao Y. CD27 and CD70 in T cell and B cell activation. Curr Opin Immunol. 2005;17(3):275–81. 10.1016/j.coi.2005.04.004 . 15886117

[59] Keller AM, Schildknecht A, Xiao Y, van den Broek M, Borst J. Expression of costimulatory ligand CD70 on steady-state dendritic cells breaks CD8+ T cell tolerance and permits effective immunity. Immunity. 2008;29(6):934–46. 10.1016/j.immuni.2008.10.009 . 19062317

[60] Simpson TR, Quezada SA, Allison JP. Regulation of CD4 T cell activation and effector function by inducible costimulator (ICOS). Curr Opin Immunol. 2010;22(3):326–32. 10.1016/j.coi.2010.01.001 . 20116985

[61] Tourneur L, Chiocchia G. FADD: a regulator of life and death. Trends Immunol. 2010;31(7):260–9. 10.1016/j.it.2010.05.005 . 20576468

[62] Elgueta R, Benson MJ, de Vries VC, Wasiuk A, Guo Y, Noelle RJ. Molecular mechanism and function of CD40/CD40L engagement in the immune system. Immunol Rev. 2009;229(1):152–72. 10.1111/j.1600-065X.2009.00782.x 19426221 PMC3826168

[63] Iwakura Y, Ishigame H, Saijo S, Nakae S. Functional specialization of interleukin-17 family members. Immunity. 2011;34(2):149–62. 10.1016/j.immuni.2011.02.012 . 21349428

[64] Sarre C, Gonzalez-Hernandez A, Van Coppernolle S, Grit R, Grauwet K, Van Meulder F, et al Comparative immune responses against *Psoroptes ovis* in two cattle breeds with different susceptibility to mange. Vet Res. 2015;46:131 10.1186/s13567-015-0277-x 26582546 PMC4652412

[65] Chiricozzi A. Pathogenic role of IL-17 in psoriasis and psoriatic arthritis. Actas Dermosifiliogr. 2014;105 Suppl 1:9–20. 10.1016/S0001-7310(14)70014-6 . 25398488

[66] Gaublomme JT, Yosef N, Lee Y, Gertner RS, Yang LV, Wu C, et al Single-Cell Genomics Unveils Critical Regulators of Th17 Cell Pathogenicity. Cell. 2015;163(6):1400–12. 10.1016/j.cell.2015.11.009 26607794 PMC4671824

[67] Wang C, Yosef N, Gaublomme J, Wu C, Lee Y, Clish CB, et al CD5L/AIM Regulates Lipid Biosynthesis and Restrains Th17 Cell Pathogenicity. Cell. 2015;163(6):1413–27. 10.1016/j.cell.2015.10.068 26607793 PMC4671820

[68] Barron L, Wynn TA. Macrophage activation governs schistosomiasis-induced inflammation and fibrosis. Eur J Immunol. 2011;41(9):2509–14. 10.1002/eji.201141869 21952807 PMC3408543

[69] Getaneh A, Tamrat A, Tadesse K. Arginase activity in peripheral blood of patients with intestinal schistosomiasis, Wonji, Central Ethiopia. Parasite Immunol. 2015;37(7):380–3. 10.1111/pim.12186 . 25786588

[70] Wu H, Zhen Y, Ma Z, Li H, Yu J, Xu ZG, et al Arginase-1-dependent promotion of TH17 differentiation and disease progression by MDSCs in systemic lupus erythematosus. Sci Transl Med. 2016;8(331):331ra40 10.1126/scitranslmed.aae0482 27009269 PMC4895207

[71] Liao SC, Cheng YC, Wang YC, Wang CW, Yang SM, Yu CK, et al IL-19 induced Th2 cytokines and was up-regulated in asthma patients. J Immunol. 2004;173(11):6712–8. 10.4049/jimmunol.173.11.6712 . 15557163

[72] Witte E, Kokolakis G, Witte K, Philipp S, Doecke WD, Babel N, et al IL-19 is a component of the pathogenetic IL-23/IL-17 cascade in psoriasis. J Invest Dermatol. 2014;134(11):2757–67. 10.1038/jid.2014.308 . 25046339

[73] Sabat R, Wallace E, Endesfelder S, Wolk K. IL-19 and IL-20: two novel cytokines with importance in inflammatory diseases. Expert Opin Ther Targets. 2007;11(5):601–12. 10.1517/14728222.11.5.601 . 17465720

[74] Goyette J, Geczy CL. Inflammation-associated S100 proteins: new mechanisms that regulate function. Amino Acids. 2011;41(4):821–42. 10.1007/s00726-010-0528-0 . 20213444

[75] Broome AM, Ryan D, Eckert RL. S100 protein subcellular localization during epidermal differentiation and psoriasis. J Histochem Cytochem. 2003;51(5):675–85. 10.1177/002215540305100513 12704215 PMC3785113

[76] Glaser R, Meyer-Hoffert U, Harder J, Cordes J, Wittersheim M, Kobliakova J, et al The antimicrobial protein psoriasin (S100A7) is upregulated in atopic dermatitis and after experimental skin barrier disruption. J Invest Dermatol. 2009;129(3):641–9. 10.1038/jid.2008.268 . 18754038

[77] Benoit S, Toksoy A, Ahlmann M, Schmidt M, Sunderkotter C, Foell D, et al Elevated serum levels of calcium-binding S100 proteins A8 and A9 reflect disease activity and abnormal differentiation of keratinocytes in psoriasis. Br J Dermatol. 2006;155(1):62–6. 10.1111/j.1365-2133.2006.07198.x . 16792753

[78] Burgess ST, Frew D, Nunn F, Watkins CA, McNeilly TN, Nisbet AJ, et al Transcriptomic analysis of the temporal host response to skin infestation with the ectoparasitic mite *Psoroptes ovis*. BMC Genomics. 2010;11:624 10.1186/1471-2164-11-624 21067579 PMC3091762

[79] Gottschalk GM, Kidson SH. Molecular analysis of vitiligo lesions reveals sporadic melanocyte survival. Int J Dermatol. 2007;46(3):268–72. 10.1111/j.1365-4632.2006.03000.x . 17343582

[80] Coda AB, Icen M, Smith JR, Sinha AA. Global transcriptional analysis of psoriatic skin and blood confirms known disease-associated pathways and highlights novel genomic "hot spots" for differentially expressed genes. Genomics. 2012;100(1):18–26. 10.1016/j.ygeno.2012.05.004 . 22584065

[81] Suarez-Farinas M, Lowes MA, Zaba LC, Krueger JG. Evaluation of the psoriasis transcriptome across different studies by gene set enrichment analysis (GSEA). PLoS One. 2010;5(4):e10247 10.1371/journal.pone.0010247 20422035 PMC2857878

[82] Beroukhim K, Danesh MJ, Nguyen C, Austin A, Koo J, Levin E. Anti-IL-23 Phase II Data for Psoriasis: A Review. J Drugs Dermatol. 2015;14(10):1093–6. . 26461819

[83] Campa M, Mansouri B, Warren R, Menter A. A Review of Biologic Therapies Targeting IL-23 and IL-17 for Use in Moderate-to-Severe Plaque Psoriasis. Dermatol Ther (Heidelb). 2016;6(1):1–12. 10.1007/s13555-015-0092-3 26714681 PMC4799039

[84] Munder M. Arginase: an emerging key player in the mammalian immune system. Br J Pharmacol. 2009;158(3):638–51. 10.1111/j.1476-5381.2009.00291.x 19764983 PMC2765586

[85] Rodriguez PC, Ochoa AC, Al-Khami AA. Arginine Metabolism in Myeloid Cells Shapes Innate and Adaptive Immunity. Front Immunol. 2017;8:93 10.3389/fimmu.2017.00093 28223985 PMC5293781

[86] Okada Y, Wu D, Trynka G, Raj T, Terao C, Ikari K, et al Genetics of rheumatoid arthritis contributes to biology and drug discovery. Nature. 2014;506(7488):376–81. 10.1038/nature12873 24390342 PMC3944098

[87] Qian X, Gao Y, Ye X, Lu M. Association of STAT6 variants with asthma risk: a systematic review and meta-analysis. Hum Immunol. 2014;75(8):847–53. 10.1016/j.humimm.2014.06.007 24952213

[88] Summerfield A, Meurens F, Ricklin M. The immunology of the porcine skin and its value as a model for human skin. Molecular Immnology. 2015; 66(1): 14–21. 10.1016/j.molimm.2014.10.023. 10.1016/j.molimm.2014.10.023 25466611

