# Supplementary material for: Early immune suppression leads to uncontrolled mite proliferation and potent host inflammatory responses in a porcine model of crusted versus ordinary scabies
Source: PLoS Negl Trop Dis. 2020 Sep 4;14(9):e0008601. doi: 10.1371/journal.pntd.0008601 (PMC7508399; doi:10.1371/journal.pntd.0008601)
Supplement: S1 Fig — Each row represents a single sample. (DOCX) [file pntd.0008601.s001.docx]

**
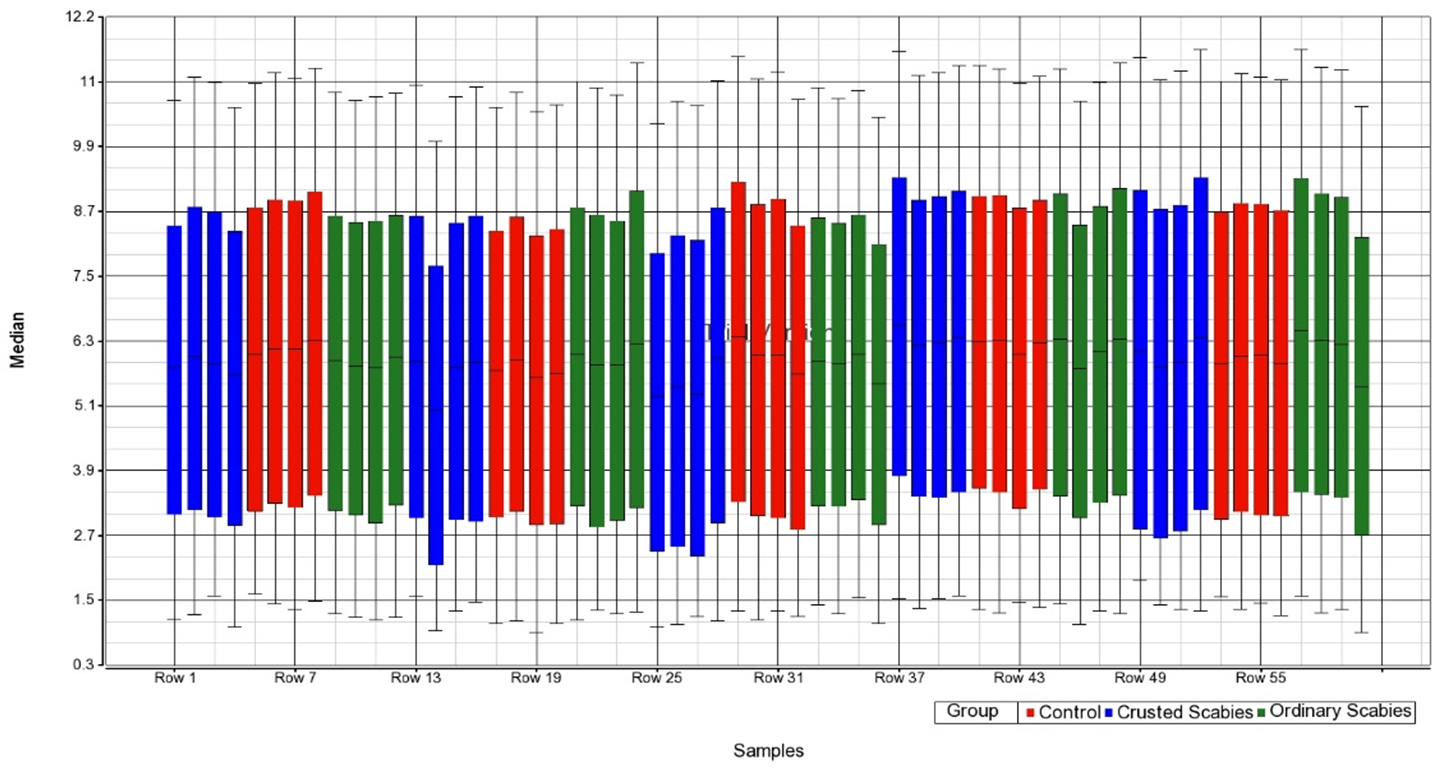
**

**S1 Figure.** Box and whisker profile plot to examine the feature intensity distributions of microarray data over the time course of infestation with *S. scabiei*. Each row represents a single sample
