# Supplementary material for: Early immune suppression leads to uncontrolled mite proliferation and potent host inflammatory responses in a porcine model of crusted versus ordinary scabies
Source: PLoS Negl Trop Dis. 2020 Sep 4;14(9):e0008601. doi: 10.1371/journal.pntd.0008601 (PMC7508399; doi:10.1371/journal.pntd.0008601)
Supplement: S2 Fig — The differentially expressed genes included are those which showed ≥ ± 2 fold change in expression with p < 0.05. Nodes coloured by gene expression with red (higher levels of expression) and pink nodes representing up regulated genes. (DOCX) [file pntd.0008601.s002.docx]

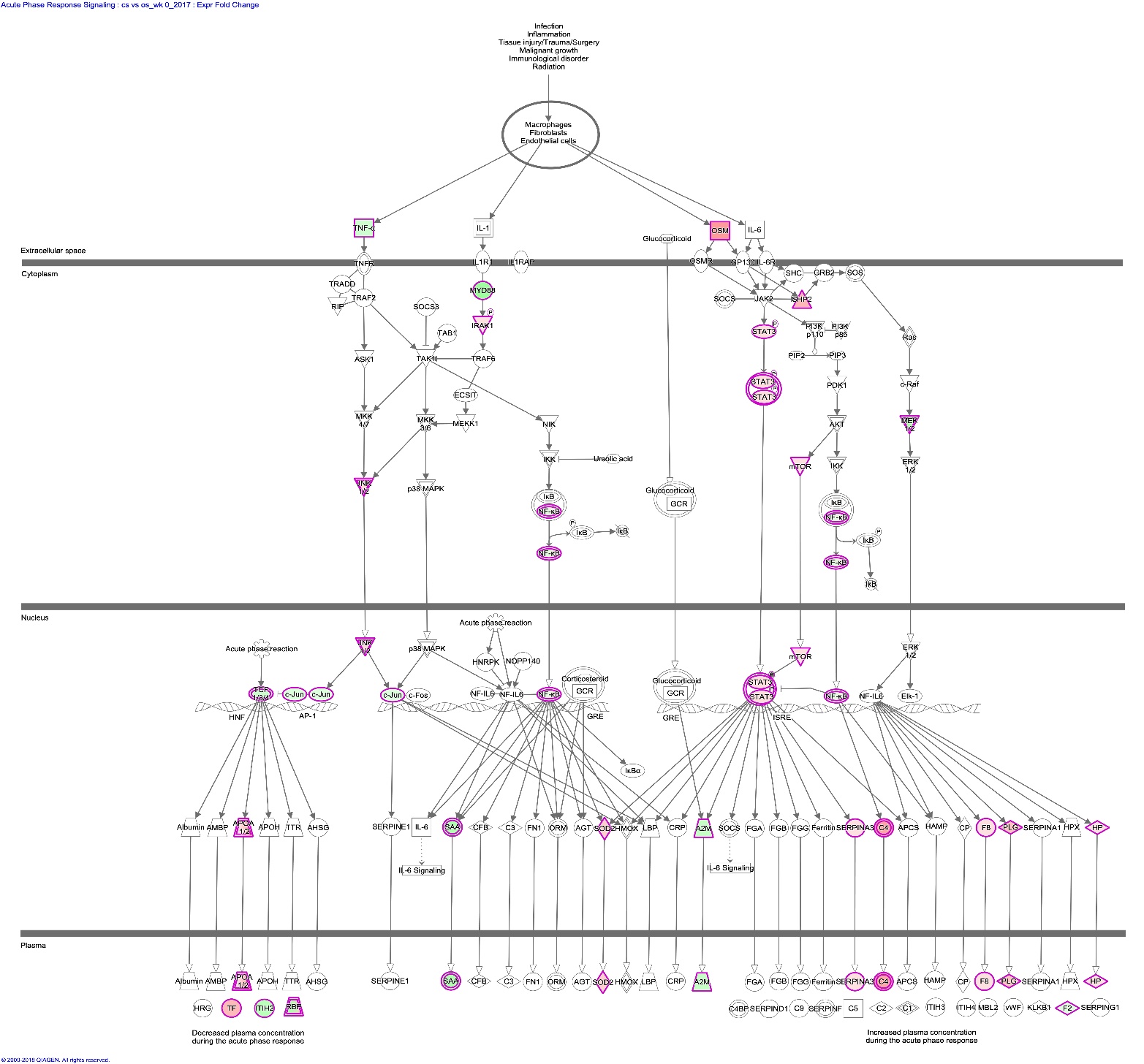


**S2 Figure. IPA canonical pathway depicting relationships among genes associated with the Acute Phase signaling pathway in CS vs OS prior to experimental infestation.** The differentially expressed genes included are those which showed ≥ ± 2 fold change in expression with p < 0.05. Nodes coloured by gene expression with red (higher levels of expression) and pink nodes representing up regulated genes.
