# Supplementary material for: Early immune suppression leads to uncontrolled mite proliferation and potent host inflammatory responses in a porcine model of crusted versus ordinary scabies
Source: PLoS Negl Trop Dis. 2020 Sep 4;14(9):e0008601. doi: 10.1371/journal.pntd.0008601 (PMC7508399; doi:10.1371/journal.pntd.0008601)
Supplement: S1 Table. Primer sequences and amplicon details for porcine qRT-PCR — (DOCX) [file pntd.0008601.s003.docx]

**S1** **Table.** Primer sequences and amplicon details for porcine qRT-PCR.

| Gene | Sequence (5’- 3’) | Size (bp) | Accession | Primer Source |
| --- | --- | --- | --- | --- |
| HPRT1 | F: GCAGCCCCAGCGTCGTGATT  R: CGAGCAAGCCGTTCAGTCCTGT | 142 | NM_001032376.2 | ([Mounsey et al., 2015](#_ENREF_109)) |
| GLO1 | F: GGATGCTCGGTTGTTCCTGT  R: GGAAGACGACTGAGCCGATT | 145 | XM_001927957.5 |  |
| FOXP3 | F: GGTGCAGTCTCTGGAACAAC  R: GGTGCCAGTGGCTACAATAC | 148 | NM_001128438.1 | ([Levast et al., 2010](#_ENREF_91)) |
| IFNG | F: CCAGGCCATTCAAAGGAGCATGGA  R: GGCTTTGCGCTGGATCTGCAGA | 140 | NM_213948.1 | ([Mounsey et al., 2015](#_ENREF_109)) |
| IL1B | F: GTGCTGGCTGGCCCACA  R: GAACACCACTTCTCTCTTCA | 71 | NM_214055 | ([Petrov et al., 2014](#_ENREF_128)) |
| NLRP3 | F: TCTGTGAGGGACTGTTGCAC  R: CGCAGGCTCTGGTTAGAAGT | 121 | NM_001256770.2 |  |
| TGFB | F: CACGGCATGAACCGGCCCTT  R: TGTAGAGCTGCCGCACGCAG | 148 | NM_214015 | ([Mounsey et al., 2015](#_ENREF_109)) |
| CD274 | F: ACCAGTTCCCAGAGAGAGGA  R: CACATATGGTTCTGGGATGACC | 150 | NM_001025221.1 |  |
| TNF | F: GCCCCCAGAAGGAAGAGTTTC  R: TCCCTCGGCTTTGACATTGG | 128 | NM_214022.1 |  |

*bp = base pair.
