# Supplementary material for: Early immune suppression leads to uncontrolled mite proliferation and potent host inflammatory responses in a porcine model of crusted versus ordinary scabies
Source: PLoS Negl Trop Dis. 2020 Sep 4;14(9):e0008601. doi: 10.1371/journal.pntd.0008601 (PMC7508399; doi:10.1371/journal.pntd.0008601)
Supplement: S2 Table — Genes that differed significantly in expression (either up or down regulated) levels upon mite infestation over the time course of 8 weeks. Gene expression was deduced by a 2- way ANOVA combined with a Fisher's Least Significant Difference (LSD) post-hoc test. These genes had a FDR corrected p-value ≤ 0.05 and fold change ≥ ± 2.0. The gene function was verified with online databases IPA, UniProt and GeneCards. The notable genes associated with signalling pathways and gene networks in the processes of immune, inflammatory and allergic responses are indicated. (DOCX) [file pntd.0008601.s004.docx]

**S2 Table.** **Differentially expressed genes in Crusted vs Ordinary scabies pigs upon infestation with *S. scabiei*.** Genes that differed significantly in expression (either up or down regulated) levels upon mite infestation over the time course of 8 weeks. Gene expression was deduced by a 2- way ANOVA combined with a Fisher's Least Significant Difference (LSD) post-hoc test. These genes had a FDR corrected p-value ≤ 0.05 and fold change ≥ ± 2.0. The gene function was verified with online databases IPA, UniProt and GeneCards. The notable genes associated with signalling pathways and gene networks in the processes of immune, inflammatory and allergic responses are indicated.

| Gene | Gene Description | Pre-infestation | Early | | Late | |
| --- | --- | --- | --- | --- | --- | --- |
|  |  | Week 0 | 1wpi | 2wpi | 4wpi | 8wpi |
|  |  | FC | FC | FC | FC | FC |
|  | **Pathogen recognition, cell signalling and transcription – receptors and factors** |  |  |  |  |  |
| TLR2 | Toll-like receptor 2 | 2.2 |  | -4.17 |  |  |
| TLR3 | Toll-like receptor 3 |  | -2.09 |  |  |  |
| TLR4 | Toll-like receptor 4 | 2.4 |  |  |  |  |
| TLR5 | Toll-like receptor 5 |  |  | -3.11 |  |  |
| TLR8 | Toll-like receptor 8 |  | 44.47 |  | 2.84 |  |
| TLR9 | Toll-like receptor 9 |  | -3.18 |  |  |  |
| JAK2 | Janus kinase 2 |  | -2.52 | -2.03 |  |  |
| STAT1 | Signal transducer and activator of transcription 1 |  |  |  | 3.77 |  |
| STAT2 | Signal transducer and activator of transcription 2 |  | -2.56 |  |  |  |
| STAT3 | Signal transducer and activator of transcription 3 | 4.58 |  | -3.55 |  |  |
| STAT4 | Signal transducer and activator of transcription 4 |  |  | 2.69 |  |  |
| STAT5A | Signal transducer and activator of transcription 5A |  | -2.54 |  |  |  |
| STAT6 | Signal transducer and activator of transcription 6 |  | -2.58 |  |  |  |
| MYD88 | Myeloid differentiation primary response 88 | -6.65 | -9.71 | 3.92 |  |  |
|  | **Pro-inflammatory molecules** |  |  |  |  |  |
| IFNA4 | Interferon alpha 4 |  |  | -2.99 |  |  |
| IFNG | Interferon gamma | 7.46 |  | -4.86 |  | 2.19 |
| IL1B | Interleukin 1 beta |  | -6.01 |  |  |  |
| IL8/CXCL8 | Interleukin 8/C-X-C motif chemokine ligand 8 |  |  |  |  | 13.9 |
| IL8R/CXCR2 | Interleukin 8 receptor/C-X-C motif chemokine receptor 2 |  |  |  |  | 4.32 |
| IL12B | Interleukin 12 subunit beta | -2.66 |  | -2.44 |  |  |
| IL12RB1 | Interleukin 12 receptor subunit beta 1 |  |  |  |  | 2.31 |
| IL12RB2 | Interleukin 12 receptor subunit beta 2 | 3.41 |  | -2.75 |  |  |
| IL13 | Interleukin 13 |  |  |  |  | 2.36 |
| IL13RA2 | Interleukin 13 receptor subunit alpha 2 |  |  |  |  | 5.05 |
| IL17A | Interleukin 17A |  |  |  |  | 4.68 |
| IL17F | Interleukin 17F | -4.26 |  |  |  |  |
| IL17RB | Interleukin 17 receptor B |  |  |  |  | 4.89 |
| IL19 | Interleukin 19 | -2.78 | -7.16 |  |  | 7.89 |
| IL20 | Interleukin 20 |  |  |  |  | 9.06 |
| NFKB2 | Nuclear factor kappa B subunit 2 | 2.54 | -2.61 | -2.93 |  | -2.19 |
| TNF | Tumour necrosis factor | -3.34 |  |  |  |  |
| OSM | Oncostatin M | 13.78 |  | -6.68 |  | 3.42 |
|  | **Acute phase related molecules** |  |  |  |  |  |
| IL6R | Interleukin 6 receptor |  |  | -3.35 |  |  |
| ALB | Albumin |  | -4.29 |  |  | -2.31 |
| CRP | C-reactive protein |  |  | -2.32 |  |  |
| CP | Ceruloplasmin |  |  | -8.74 | 4.16 |  |
| HP | Haptoglobin | 2.58 |  | -3.13 |  |  |
| TF | Transferrin | 9.68 |  | -3.51 |  |  |
| SAA1 | Serum amyloid A1 | -5.44 | -3.02 | 2.67 | 2.23 | -2.58 |
|  | **Complement components** |  |  |  |  |  |
| C1QA | Complement C1q A chain | -2.68 |  |  |  | -3.68 |
| C3 | Complement C3 |  | -2.09 | -2.36 |  | -2.63 |
| C4B | Complement C4B | 12.36 |  | -4.48 |  | 2.39 |
| CFD | Complement factor D |  |  | -2.43 |  | -2.66 |
| C6 | Complement C6 | 6.78 | 5.01 | -9.13 |  |  |
| C8G | Complement C8 gamma chain |  |  | -2.85 |  |  |
|  | **Immunoregulatory molecules** |  |  |  |  |  |
| FOXP3 | Forkhead Box P3 |  | 3.11 | -3.85 |  |  |
| IL27 | Interleukin 27 | 4.31 |  | -5.91 |  |  |
| TGFB1 | Transforming growth factor beta 1 |  | -4.31 |  |  | 2.17 |
| CD274/PDL1 | CD274 molecule / Programmed death ligand 1 | -5.75 | -5.02 | 4.08 |  |  |
|  | **Cell surface receptors and ligands** |  |  |  |  |  |
| CD1A | CD1A molecule |  |  | -2.52 |  |  |
| CD1B | CD1B molecule |  | -8.06 |  |  |  |
| CD1D | CD1D molecule |  |  | -3.63 |  |  |
| CD1E | CD1E molecule |  | -3.6 | 3.04 |  |  |
| CD3E | CD3 molecule, epsilon |  |  |  |  | 3.44 |
| CD3G | CD3 gamma molecule |  | 20.56 |  | 8.86 | 3.57 |
| CD5L | CD5 antigen-like |  | -4.36 | -3.81 |  |  |
| CD40LG | CD40 ligand |  |  |  |  | 3.59 |
| CD82 | CD82 molecule |  | -3.1 |  |  |  |
| CD86 | CD86 molecule |  |  | -2.9 |  | -3.32 |
| CD70/TNFSF7 | CD70 molecule | -5.04 | -8.21 | 3.24 |  |  |
| CD4 | CD4 molecule |  | -2.11 |  |  | 2.02 |
| CD8A | CD8 alpha molecule | -3.42 | -3.05 | 2.94 |  |  |
|  | **Chemokines- receptors and ligands** |  |  |  |  |  |
| CCL4 | C-C motif chemokine ligand 4 |  |  |  |  | 2.36 |
| CCL5 | C-C motif chemokine ligand 5 |  |  | -2.73 |  |  |
| CCL17/TARC | C-C motif chemokine ligand 17/Thymus and activation-regulated chemokine |  |  |  |  | 2.38 |
| CCL20 | C-C motif chemokine ligand 20 |  |  |  |  | 9.05 |
| CCL27/CTACK | Chemokine (C-C motif) ligand 27/Cutaneous T-cell activating chemokine |  |  |  |  | 3.24 |
| CCR7 | C-C motif chemokine receptor 7 |  |  |  |  | 4.59 |
| CCR10/GPR2 | C-C motif chemokine receptor 10/ G protein-coupled receptor-2 | 4.04 |  | -2.27 |  |  |
| CXCL2 | C-X-C motif chemokine ligand 2 |  |  |  |  | 6.14 |
| CXCL6 | C-X-C motif chemokine ligand 6 |  |  |  |  | 64.32 |
| ICAM3 | Intercellular adhesion molecule 3 | 2.93 |  | -6.2 |  | 3.32 |
| SELPLG | Selectin P ligand | 8.61 |  | -4.08 |  | 3.91 |
|  | **S100s** |  |  |  |  |  |
| S100A7 | S100 calcium binding protein A7 (Psoriasin 1) |  |  |  |  | 21.53 |
| S100A8 | S100 calcium binding protein A8 (Calgranulin A) |  |  |  |  | 19.86 |
| S100A9 | S100 calcium binding protein A9 (Calgranulin B) |  |  |  |  | 21.08 |
| S100A11 | S100 calcium binding protein A11 (Calgizzarin) |  | 6.69 |  |  |  |
| S100A12 | S100 calcium binding protein A12 (Calgranulin C) |  |  |  |  | 14.99 |
| S100A16 | S100 calcium binding protein A16 | 4.19 | -2.07 | -3.29 |  |  |
|  | **Miscellaneous – growth factors, enzymes, signalling molecules, etc.** |  |  |  |  |  |
| ARG1 | Arginase 1 | 8.05 |  | 7.13 |  | 30.79 |
| ARG2 | Arginase 2 |  | -2.39 |  |  | 4.03 |
| FCGR2B | Fc fragment of IgG receptor IIb | 3.75 | -2.49 | 2.23 | 2.4 | 2.04 |
| GZMB | Granzyme B |  |  |  |  | 3.52 |
| RFX5 | Regulatory factor X5 | 5.86 | 20.85 | -3.53 | 11.71 |  |
| CSF3 | Colony stimulating factor 3 |  |  |  |  | -2.81 |
| CSF3R | Colony stimulating factor 3 receptor |  |  | -2.38 |  | -2.73 |
| IL15 | Interleukin 15 | 3.34 | -2.73 |  |  |  |
| IL33 | Interleukin 33 |  |  | -3.06 |  |  |
| DCT | Dopachrome tautomerase | -12.32 | -33.75 | 18.12 |  | -6.40 |
| HLA/SLA-3 | MHC class I antigen 3 |  |  |  |  | 2.6 |
| HLA/SLA-5 | MHC class I antigen 5 | -4.59 | -6.1 |  |  |  |
| HLA-A | MHC class I antigen A |  |  |  | 5.13 |  |
| HLA-DOB | MHC Class II, DO beta |  | -2.18 |  |  |  |

*FC = fold change

|  |  |  |  |  |  |
| --- | --- | --- | --- | --- | --- |
|  |  |  |  |  |  |
