# Supplementary material for: Early immune suppression leads to uncontrolled mite proliferation and potent host inflammatory responses in a porcine model of crusted versus ordinary scabies
Source: PLoS Negl Trop Dis. 2020 Sep 4;14(9):e0008601. doi: 10.1371/journal.pntd.0008601 (PMC7508399; doi:10.1371/journal.pntd.0008601)
Supplement: S3 Table — These genes were identified in the 2-way ANNOVA analysis with a p-value of ≤ 0.05 and FC of > 2.0. Relative quantification of gene expression levels was determined by normalising to the HPRT1 control using the comparative Ct method with the formula 2 -ΔΔCT and expressed as fold change. Duplicates of each cDNA sample from infested (OS, n = 4; CS, n = 4) and non-infested controls (C, n = 4) were maintained. Wpi = weeks post-infestation. (DOCX) [file pntd.0008601.s005.docx]

**S3 Table.** **Confirmation of eight upregulated genes in microarray data by qPCR.** These genes were identified in the 2-way ANNOVA analysis with a p-value of ≤ 0.05 and FC of > 2.0. Relative quantification of gene expression levels was determined by normalising to the HPRT1 control using the comparative Ct method with the formula 2 ^-ΔΔCT^ and expressed as fold change. Duplicates of each cDNA sample from infested (OS, n = 4; CS, n = 4) and non-infested controls (C, n = 4) were maintained. Wpi = weeks post-infestation.

| Gene Symbol | Gene Description | qRT-PCR validation  fold change | Microarray analysis  fold change | Comparison | Time  (wpi) |
| --- | --- | --- | --- | --- | --- |
| IFNG | Interferon gamma | 3.64 | 2.40 | CS vs C | 1 |
| IL1B | Interleukin 1 beta | 4.50 | 2.59 | OS vs C | 1 |
| FOXP3 | Forkhead box P3 | 24.95 | 3.29 | CS vs C | 2 |
| GLO1 | Glyoxalase 1 | 6.29 | 7.93 | CS vs OS | 4 |
| TGFB | Transforming growth factor beta | 3.32 | 2.17 | CS vs OS | 8 |
| CD274 | Cluster of differentiation 274 | 3.40 | 4.37 | OS VS C | 8 |
| NLRP3 | NOD like receptor protein 3 | 2.60 | 2.44 | OS vs C | 8 |
| TNF | Tumour necrosis factor | 4.84 | 2.45 | OS vs C | 8 |
